# Geraniol-a potential alternative to antibiotics for bovine mastitis treatment without disturbing the host microbial community or causing drug residues and resistance

**DOI:** 10.3389/fcimb.2023.1126409

**Published:** 2023-02-16

**Authors:** Wei Guo, Min Qiu, Zhonghui Pu, Nana Long, Min Yang, Ke Ren, Ruihong Ning, Siyuan Zhang, Fu Peng, Fenghui Sun, Min Dai

**Affiliations:** ^1^ School of Laboratory Medicine, Chengdu Medical College, Chengdu, China; ^2^ Sichuan Provincial Engineering Laboratory for Prevention and Control Technology of Veterinary Drug Residue in Animal-origin Food, Chengdu Medical College, Chengdu, China; ^3^ West China School of Pharmacy, Sichuan University, Chengdu, China

**Keywords:** mastitis, geraniol, gut microbiome, antibacterial, anti-inammatory, residue, drug resistance

## Abstract

Mastitis is one of the most prevalent diseases of dairy cows. Currently, mastitis treatment in dairy cows is mainly based on antibiotics. However, the use of antibiotics causes adverse effects, including drug resistance, drug residues, host-microbiome destruction, and environmental pollution. The present study sought to investigate the potentiality of geraniol as an alternative to antibiotics for bovine mastitis treatment in dairy cows. Additionally, the effectiveness of treatment, improvement in inflammatory factors, the influence on microbiome, presence of drug residues, and drug resistance induction were compared and analyzed comprehensively.Geraniol showed an equivalent therapeutic rate as antibiotics in the mouse infection model and cows with mastitis. Moreover, geraniol significantly inhibited the pathogenic bacteria and restored the microbial community while increasing the abundance of probiotics in milk. Notably, geraniol did not destroy the gut microbial communities in cows and mice, whereas antibiotics significantly reduced the diversity and destroyed the gut microbial community structure. Additionally, no geraniol residue was detected in milk four days after treatment discontinuation, but, antibiotic residues were detected in milk at the 7th day after drug withdrawal. In vitro experiments revealed that geraniol did not induce drug resistance in the Escherichia coli strain ATCC25922 and Staphylococcus aureus strain ATCC25923 after 150 generations of culturing, while antibiotics induced resistance after 10 generations. These results suggest that geraniol has antibacterial and anti-inflammatory effects similar to antibiotics without affecting the host-microbial community structure or causing drug residues and resistance. Therefore, geraniol can be a potential substitute for antibiotics to treat mastitis or other infectious diseases and be widely used in the dairy industry.

## Introduction

Bovine mastitis, a fatal mammary gland infection, is the most common disease of dairy cattle affecting the global dairy industry (Rollin et al., 2015; Dahl et al., 2018). Mastitis is mainly caused by mechanical injury, pathogen infection, and decreased of immunity, etc (Kibebew, 2017). The invasion of pathogens, such as *Escherichia coli* (Ganda et al., 2017), *Staphylococcus aureus* (Naushad et al.), *Streptococcus agalactiae* (Keefe, 1997), and *Mycoplasma* (Geary et al., 1981) induces bacterial mastitis in cows. Currently, antibiotics are the only treatment option available for bacterial mastitis in dairy cows (Guardabassi et al., 2018). However, the use and abuse of antibiotics in animal husbandry have significantly increased antibiotic resistance globally (Pal et al., 2016), posting a serve threat to public health (Shankar, 2014). Besides, antibiotic residues have been detected in cow’s milk following antibiotic treatment (Liljebjelke et al., 2000). Furthermore, the use of antibiotics disrupts the host-microbial community (Cho et al., 2012; Cox et al., 2014; Kuperman and Koren, 2016). For instance, the antibiotics killing the mastitis-causing bacteria also damage the microbial diversity (Ganda et al., 2016), adversely affecting the host immunity, defense, nutrient absorption, and overall health (Kamada et al., 2013; Porcellato et al., 2020). Therefore, finding a safe, reliable, and efficient drug alternative to antibiotics for mastitis treatment has great significance.

In recent years, natural medicines have attracted attention in the field of drug research due to their advantages of broad-spectrum antibacterial activity (Long NN et al., 2020; Lin et al., 2021), immune regulation, low drug residues, low toxicity, and high resistance (Harvey et al., 2015). As such, several natural Chinese herbs have been screened for antibacterial activity, of which *Fructus Tsaoko* (a traditional Chinese Medicine, TCM) essential oil showed significant *in vivo* and *in vitro* antibacterial activities (Min et al., 2016; Sun et al., 2022). Geraniol and 1, 8-cineole are the major components of *Fructus Tsaoko* essential oil and play a key vital in defending the host from bacterial infection (Min et al., 2016). It is reported that geraniol poses several pharmacological activities, such as analgesic, antibacterial, anti-inflammatory, antioxidant, anti-ulcer, antifungal, and insecticidal effects (Solórzano-Santos and Miranda-Novales, 2011; Guimarães et al., 2013; Medicherla et al., 2015; Lei et al., 2019; Younis et al., 2020), of which the antitumor activity has attracted increasing attention (Carnesecchi et al., 2004; Cho et al., 2016; Lei et al., 2019; Mączka et al., 2020). Besides, geraniol exerts protective effects on the liver, heart, and nerves (Lei et al., 2019; Junior et al., 2020; Mączka et al., 2020) and regulates apoptosis (Zhang et al., 2019; Junior et al., 2020). Therefore, geraniol could be used as an active ingredient and a chemical adjuvant for drug development.

TCM has been widely used to treat various diseases by regulating the gut microbiota structure (Tong et al., 2018; Pan et al., 2020; Xu et al., 2020). The mechanism of action of TCM includes two aspects: inhibiting harmful bacteria and protecting beneficial bacteria (Tang et al., 2018; Pan et al., 2020). Unlike antibiotics, TCM interfere with the host-microbial community and restores the bacterial community structure destroyed by antibiotics during disease treatment (Xu et al., 2014; Yu et al., 2017). In a previous study, geraniol showed excellent *in vitro* and *in vivo* antibacterial activities against strains isolated from clinical bovine mastitis samples (Aiemsaard et al., 2011), such as *Escherichia coli*, *Streptococcus agalactiae*, *Staphylococcus aureus*, and *Bacillus cereus*, demonstrating its potentiality as an alternative to antibiotics in treating clinical mastitis of dairy cows. Therefore, in this study, geraniol was used to treat clinical mastitis using antibiotics as the control, and, several parameters, including the effectiveness of the treatment, the effect of the medicine on the host microbial community, drug residues in milk, and drug resistance in bacteria, were analyzed to explore the possibility of geraniol as an antibiotic substitute for bacterial infectious diseases.

## Materials and methods

### Materials involved in the study

Geraniol (purity > 98%), was obtained from Sigma Chemical Co. (St. Louis, MO), and its solubility was improved by emulsifying in anhydrous glycerol according to the previously reported method (Pavan et al., 2018). Specific pathogen-free (SPF) Kunming (KM) mice (male, body weight, 20 ± 2 g) were provided by Sichuan Chengdu Institute of Biological Products Co., Ltd. (Chengdu, China). The *Escherichia coli* strain ATCC25922 was obtained from the American Type Culture Collection (ATCC, Rockville, MD, USA). The geraniol standard (purity > 98%, No. SHBL2152), HPLC-grade acetonitrile (ACN), and methanol (MeOH) were purchased from Sigma-Aldrich (St. Louis, MO, USA). Cephalexin (purity > 98%, No. 130408-201411) and kanamycin (purity > 98%, No. 130556-201502) were obtained from the National Institute for Food and Drug Control (Beijing, China).

### Mouse model of *E. coli* infection

A preliminary experiment was conducted in mice to evaluate its efficacy, safety, and dosage before using geraniol to treat mice infected with *Escherichia coli* strain ATCC25922. After adaptive feeding for two weeks in the laboratory, 80 KM mice were randomly divided into eight groups (n = 10), including seven experimental groups and one blank group. The *Escherichia coli* strain ATCC25922 was cultured at a constant temperature of 37°C in Luria-Bertani (LB) medium (50 mL) until the culture reached a logarithmic phase.

Then, the bacterial culture was diluted to seven concentrations (5.85 × 10^7^, 2.925 × 10^7^, 3.663 × 10^6^, 2.75 × 10^6^, 1.831 × 10^6^, 1.371 × 10^6^, and 0.911 × 10^6^ CFU/mL) using PBS and mixed with 5% porcine stomach mucin (Sigma, St Louis, MO, USA).

The mouse model with *E. coli* infection was successfully established following the previously study (Long et al., 2022). The minimum concentration causing the death of all and half of the mice in a group was considered as the minimum lethal dose (MLD) and half lethal dose (LD_50_) of *E. coli*, separately.

Afterwards, 90 KM mice were randomly divided into nine groups (n = 10), including the blank, *E. coli* infection, solvent, positive control, and five geraniol treatment groups. The blank and *E. coli* infection groups were intramuscularly injected with PBS; the solvent group was intramuscularly injected with Twain-80; the positive control group was intramuscularly injected with cefotaxime (0.3 g/kg); and the geraniol treatment groups were intramuscularly injected with different doses of geraniol (0.261 g/kg, 0.166 g/kg, 0.092 g/kg, 0.07 g/kg, and 0.045 g/kg). Each mouse was injected once daily for 5 days. After the last intramuscular injection, the mice in all groups, except for the blank group, were intraperitoneally injected with the MLD of *E. coli*. The survival rate of the mice was observed 7 days after *E. coli* challenge, and the dose of geraniol maintaining 50% survival rate was considered as ED_50_.

### Assessment of antibacterial activity of geraniol in a mouse infection model

The efficacy of geraniol was evaluated by treating the mice infected with LD_50_ of *E. coli* with the ED_50_ of geraniol. 40 KM mice were randomly divided into four groups (n = 10), including the control, half-lethal *E. coli* infection, geraniol treatment, and antibiotic treatment groups. The half-lethal *E. coli* infection, antibiotic treatment, and geraniol treatment groups were challenged with the LD_50_ of *E. coli* and then intramuscularly injected with physiological saline, cefotaxime (0.3 g/kg), and geraniol (ED_50_) once a day for five consecutive days, respectively. The control group was challenged with the same dose of inactivated *E. coli*, and intramuscularly injected with physiological saline.

Later, the blood was collected from the eyeball venous plexus of mice on 0.5d, 1d, 2d, 3d, and 7d after infection, and the blood parameters were determined using TEK-II MINI A3-0052 three-class blood cell counter (Tekang Technology, China). All the experiments were completed within 24 h after blood collection. The fecal samples were collected before (0 h) and after (12 h) *E. coli* infection to explore the effect of infectious pathogens on the mouse gut microbiome. The samples collected before infection were used as a baseline (0 day) of the mouse gut microbiome. The fecal samples were collected on the 1^st^, 7^th^, 14^th^, and 28^th^ days after treatment. All samples were quickly frozen in liquid nitrogen and transferred to a -80°C freezer until further analysis. The schematic diagram of this experiment is illustrated in **Figure S1A**.

### Oral administration of geraniol in healthy mice

Theeffect of oral geraniol on gut microbiome community structure was analyzed. 30 KM mice were divided into three groups (n = 10), including the control, antibiotic, and geraniol groups. The mice of the antibiotic and geraniol groups were fed with the therapeutic dose of cefotaxime (0.3 g/kg) and geraniol (0.261 g/kg), respectively, by gavage from 1st to 3rd day, once a day. The mice of the control group were fed with physiological saline by gavage. The fecal samples were collected from all mice on days 0, 1, 3, 7, 14, and 28. The schematic diagram of this experiment is illustrated in **Figure S1B**.

### Experimental set-up in dairy cows

This study was conducted on a dairy farm in Chengdu, Sichuan Province, China, from April 2020 to September 2020. All cows used in this study were imported Holstein lactating adult cows (3–6 years) from Uruguay. The cows were provided with a standard diet composed of grass-legume hay to meet the daily nutrient requirements for milk production. The cows did not receive antibiotics or other drugs during the past two months. The somatic cell count (SCC) and clinical symptoms were used to diagnose mastitis (Pilla et al., 2013). The SSC in milk was measured using a Bentley FTS/FCM400 Combi Instrument (Chaska, USA). A total of 12 healthy and 27 infected (with subacute clinical mastitis) cows were recruited for this study. The mastitis group was divided into the antibiotic treatment (n = 14) and the geraniol treatment groups (n = 13). The drug was injected into the cows’ breasts. Detailed information on individual cows and grouping is presented in Table S1.

### Mastitis treatment and sample collection

A drug dosage of 0.0944 g/kg was considered as the ED50 of geraniol against *E. coli* infection in mice. The dosage per unit body weight of cow was about 1/24 of the mice. Therefore, the dosage of geraniol administered to cows was about 0.00393 g/kg. If the average weight of cows is around 600 kg, the dosage of each cow is 2.36 g. In the pre-experiment, high (2.36 g), medium (1.18 g), and low (0.59 g) dosage of geraniol were administered, respectively. The effects of high and medium dosages were relatively good. Therefore, the medium dosage (1.18 g) was selected to treat the cows in this study.

In the geraniol treatment group, each cow was injected with 1.18 g of geraniol twice a day. In the antibiotic treatment group, each cow was injected with 10 g of cefalexin and kanamycin monosulfate intramammary infusion (Cephalexin: Kanamycin = 2:1; Tullyvin, Cootehill, Co. Cavan, Ireland) once a day. The treatments were carried out for 5 consecutive days. The cows with mastitis with no significant improvement after 7 days of treatment were considered incurable by the drug. The fresh stool, milk, and blood samples collected on 0 days were considered baseline and sampled on the 3^rd^, 5^th^, and 14^th^ days. The middle part of the fresh stool was collected immediately after defecation using a sterile fecal collector. The nipples were wiped with iodine and 75% ethanol and cleaned using sterile distilled water to collect the unpolluted milk. During sampling, the first three milk strands were discarded, and then the milk (50 mL) was squeezed into a sterile centrifuge tube. The venous blood was obtained using a sterile syringe and centrifuged at 3000 rpm for 5 min to obtain the serum. The serum samples were stored at -4°C, while the feces and milk samples were stored in a -80°C freezer until further analysis. The schematic diagram of this experiment is illustrated in **Figure S1C**.

### Detection of inflammatory cytokines in cows

The levels of interleukin-6 (IL-6), interleukin-1β (IL-1β), tumor necrosis factor-α (TNF-α), cyclooxygenase-2 (COX-2), and inducible nitric oxide synthase (iNOS) in the serum of dairy cows with clinical mastitis were measured using the enzyme-linked immunosorbent assay (ELISA) kits (Shanghai Future Industrial Co., Ltd., Shanghai, China) according to the manufacturer’s instructions (Long et al., 2022).

### Bacterial DNA extraction, 16S rRNA sequencing, and bioinformatic analysis

The total bacterial genomic DNA was extracted using the Mo Bio PowerFecal DNA isolation kit (Mo Bio Laboratories, Carlsbad, CA, USA), following the manufacturer’s instructions. Later, a frozen aliquot (200 mg) of each stool sample was transferred directly to the PowerBead tubes to extract the total genomic DNA. Then, 10 mL of each milk sample was centrifuged at 12,000 g for 5 min at 4°C to remove the lipid layer. The supernatant was discarded, and the watercourse was collected and filtered through a 0.2 µm filter membrane. The filter membrane was transferred to the PowerBead tubes to extract the total bacterial genomic DNA. The concentration and purity of the genomic DNA were measured using NanoDrop (Thermo Fisher Scientific, Waltham, USA). The universal 515F/806R barcoded primer pair (Caporaso et al., 2011) was used to amplify the V4 region of the bacterial 16S rRNA gene. The sequencing libraries were constructed using the MiSeq Reagent Kit v2 (Illumina, CA, USA). PCR, sequencing library construction, and paired-end sequencing (Illumina MiSeq platform) were performed at Novogene (Beijing, China).

The 16S rRNA gene raw sequences were pre-processed and analyzed using the QIIME2 pipeline (version 2019.9) (Bolyen et al., 2018). Low quality (< Q20), chimeric, and erroneous reads were filtered by DADA2 software (Callahan et al., 2016). The amplicon sequences variants (ASVs) were generated from clean reads using the DADA2 pipeline. Later, aphylogenetic tree based on the clean reads was constructed using the FASTTREE algorithm (Price et al., 2012). The taxonomy was determined by BLAST using the representative sequences against the SILVA reference database (version 132) (Quast et al., 2013). The ASVs tables were rarefied to the minimum number of reads per sample before alpha or beta diversity analysis to eliminate the bias caused due to variant sequencing depth. The alpha-diversity indices, including the number of observed ASVs and Shannon’s diversity index, were calculated using QIIME2. PCoA (Principal Co-ordinate Analysis) was performed based on weighted UniFrac distances in QIIME2 to explore the similarity in the bacterial community between the samples (Lozupone and Knight, 2005).

### Quantitative PCR analysis of pathogenic bacteria and probiotics in milk

The quantitative PCR primers for *Enterobacteriaceae*, *Streptococcus*, *Mycoplasma*, *Lactobacillus*, and *Bifidobacterium* were designed according to the 16S rRNA representative sequences annotated as these genera. The DNA of cow milk bacteria in the antibiotic treatment group and the geraniol treatment group was used as a template for quantitative PCR.The primers and thermocycling parameters are presented in Table S2 and Table S3. Taking the bacteria in the dairy cows’ milk obtained on day 0 as the baseline, the relative quantification of *Enterobacteriaceae*, *Streptococcus*, *Mycoplasma*, *Lactobacillus*, and *Bifidobacterium* was calculated on the 3^rd^, 5^th^, and 14^th^ days. The 16S rRNA gene was used for bacterial normalization.

### Determination of drug residues in dairy cows’ milk after antibiotic or geraniol injection

After injecting antibiotics and geraniol into the mammary gland, the drug residues in milk were determined every day from the 1st to the 7th day till the discontinuation treatment. The geraniol residue in milk was detected and analyzed by gas chromatography-mass spectrometer (GC-MS; Agilent 7890A/5977B gas chromatography-mass spectrometer system) (Santa Clara, California, USA) following a previously reported method (Min et al., 2016), whereas cephalexin and kanamycin residues were analyzed by high-performance liquid chromatography (HPLC; Agilent LC-1260, Agilent Technologies, Santa Clara, CA, USA), according to the national standards GB/T 21314-2007 (Dasenaki et al., 2015) and GB/T 22969-2008 (GB/T 22969-2008, 2008), separately. Afterwards, a quarter of the antibiotic treatment dose (about 2.0 g) was injected into the cow’s (n=5) breast, and its residue in milk was detected following the above method to verify the consequence of different injection dose on longer residual time of antibiotics in milk.

### Assessment of geraniol and antibiotics induced bacterial drug resistance

The standard strain (*E. coli*, ATCC25922, and *S. aureus*, ATCC25923) was diluted into 0.5 Michaelis concentration units (1.5 × 10^8^ CFU/mL) with sterile normal saline, and then diluted 100 times with Mueller-Hinton Broth (MHB) medium. Later, a mother liquor of amoxicillin and ampicillin was prepared at a concentration of 4096 µg/mL using Tween-80 as an emulsifier. The minimum inhibitory concentration (MIC) of geraniol emulsion, amoxicillin, and ampicillin were determine by diluting their mother liquor into 12 different concentrations by double dilution. Afterwards, 1 mL of the MHB culture solution, 0.5 mL of the diluted geraniol solution, and 0.5 mL of the standard strain (1.5 × 10^8^ CFU/mL) were added to the test tube. The final concentrations of geraniol, amoxicillin, and ampicillin in the test tube were maintained from 0.02 to 43.00 mg/mL, 1 to 2048 µg/mL, and 1 to 2048 µg/mL, respectively. Later, the mixture was incubated at 37 °C for 24 h to observe the growth of the standard strain. The minimum drug concentration inhibiting the growth of the standard strain was regarded as the MIC value of the bacteria. The strains cultured in the MHB medium supplemented with sub-inhibitory concentration (1/2MIC) induction drugs at 37 °C and 200 rpm for 24 h were regarded as the first-generation resistant strains. The first-generation resistant strain was repeatedly cultured with geraniol, amoxicillin, and ampicillin to obtain the second-generation strain, and the new MIC values of geraniol, amoxicillin, and ampicillin were determined every ten generations following the above method.

### Statistical analysis

Significance differences between the mean values of three or more groups were analyzed using one-way ANOVA, followed by the *post-hoc* Dunn’s multiple comparison tests in GraphPad Prism 7 (GraphPad Software, Inc., USA). The differences in mean values between the two groups were compared using Mann-Whitney U test. The graphs were plotted using the “boxplot,” “barplot,” “ggplot2”, and “plot” functions in base R (version 3.5) (Stiglic et al., 2019).

## Results

### Mouse model of *E. coli* infection

The MLD of the mice and LD_50_ of *E. coli* were 2.925×10^7^ and 0.911×10^6^ CFU/kg, respectively (Table S4). After 5 days of prophylactic administration of geraniol, significant antibacterial activity was observed in the mice challenged with MLD of *E. coli*. Geraniol at 0.261 g/kg completely (100%) prevented *E. coli* infection. The prevention rate decreased with the decrease in dose, and the drug dose of 0.0944 g/kg was considered as the ED_50_ of geraniol against *E. coli* infected mice (Table S5).

### Therapeutic effect of geraniol on mice infected with LD_50_ of *E. coli*

**Figure 1:**
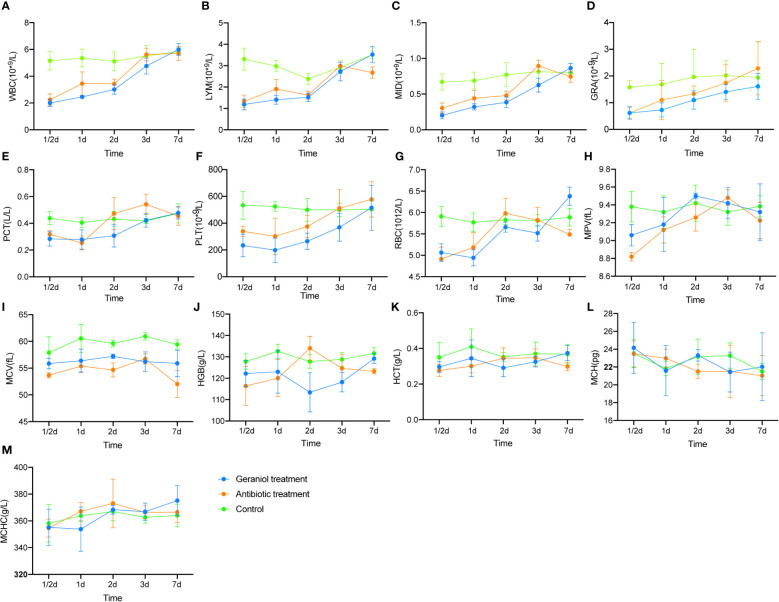
Effect of geraniol and antibiotics on the recovery of Blood Routine Indexes in mice with bacterial infection. **(A)** white blood cell count (WBC); **(B)** absolute lymphocyte value (LYM); **(C)** absolute intermediate cell (MID); **(D)** absolute granulocyte value (GRA); **(E)** platelet packed volume (PCT); **(F)** platelet count (PLT); **(G)** red blood cell count (RBC); **(H)** average platelet volume ((MPV); **(I)** mean red blood cell volume (MCV); **(J)** hemoglobin (HGB); **(K)** hematocrit (HCT); **(L)** mean hemoglobin content (MCH); **(M)** mean hemoglobin concentration (MHCH). h means hours; d means day.

Similar to cefotaxime, geraniol cured 100% of infection in the mice challenged with LD_50_ of *E. coli* (Table S6). The routine blood indices, including WBC, LYM, MID, GRA, PCT, PLT, RBC, MPV, and MCV, significantly decreased after infection (p < 0.05; **Figure S2**). These indices increased after treatment with cefotaxime and geraniol (**Figure 1**), and reached a similar level to that of the healthy mice. of them, LYM, MID, RBC, MPV, and MCV were restored to significantly higher levels in the geraniol treatment group than in the antibiotic treatment group.

### Effects of antibiotics and geraniol on gut microbiota community structure of mice during infection

**Figure 2:**
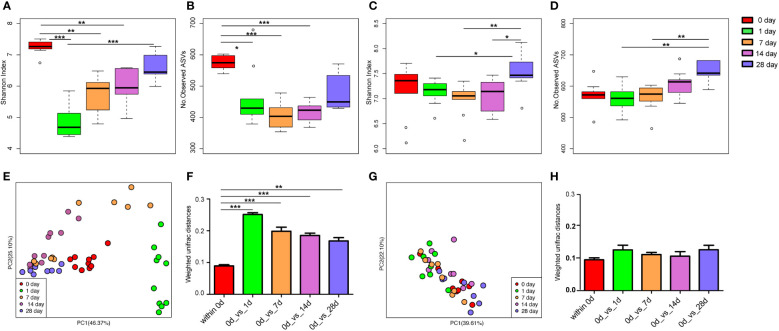
Dynamic changes of alpha and beta diversity of gut microbiotas in *Escherichia coli* (LD50) infection model mice treated with antibiotics and geraniol. Shannon index **(A)** and number of observed ASVs **(B)** of gut microbiotas in bacterial infection model mice treated with antibiotics; Shannon index **(C)** and number of observed ASVs **(D)** of gut microbiotas in bacterial infection model mice treated with geraniol; Principal coordinate analysis (PCoA) based on Weighted Unifrac distance in antibiotic treatment group **(E)** and geraniol treatment group **(G)**; Dynamic changes of phylogenetic distance of gut microbiotas of mice after administration of antibiotics **(F)**/geraniol **(H)** and before administration. 0th day indicates that the mice are infected with *E coli* and have not been treated with drugs; 1st day means 1 day after injection drug; 5th day means 5 days after continuous injection drug, and stopping administration; 14th and 28th day mean 14, 28 days after administration, separately. (p values calculated by the One-way ANOVA test followed by *post-hoc* Dunn’s multiple comparisons test: ^∗^< 0.05, ^∗∗^< 0.01 and ^∗∗∗^< 0.001).

There was no significant difference in the alpha diversity indices (Shannon index and the number of observed ASVs; **Figures S3A, B**) and PCoA (**Figure S3C**) of mice gut microbiota infected with *E. coli* than the pre-infected mice. After treatment with cefotaxime, the Shannon index (**Figure 2A**) and the number of observed ASVs (**Figure 2B**) significantly decreased. The gut microbial diversity significantly decreased on the 1^st^, 7^th^, and 14^th^ days after intraperitoneal injection of antibiotics (p < 0.05) and did not return to normal until the 28^th^ day. PCoA by the weighted UniFrac matrix suggested that antibiotic treatment destroyed the gut microbiota (**Figure 2E**). The phylogenetic distance between the gut microbiota after (1^st^/7^th^/14^th^/28^th^ day) and before treatment was significantly larger than that within the samples before treatment (the 0^th^ day) (**Figure 2F**; p < 0.05), where the gut microbiota of the mice before treatment was considered as the baseline. After geraniol treatment, the Shannon index and the number of observed ASVs of gut microbiota showed no significant decrease, while those on the 28^th^ day were significantly higher than those on the 1^st^ and 7^th^ days (p < 0.05; **Figures 2C, D**). Meanwhile, the PCoA of gut microbiota showed no significant changes after geraniol treatment (**Figure 2G**). The phylogenetic distance between the gut microbiota after (1^st^/7^th^/14^th^/28^th^ day) and before treatment was not significantly different from the distance between the gut microbiota before treatment (p > 0.05; **Figure 2H**).

### Effect of geraniol direct feeding on the gut microbiome of mice

**Figure 3:**
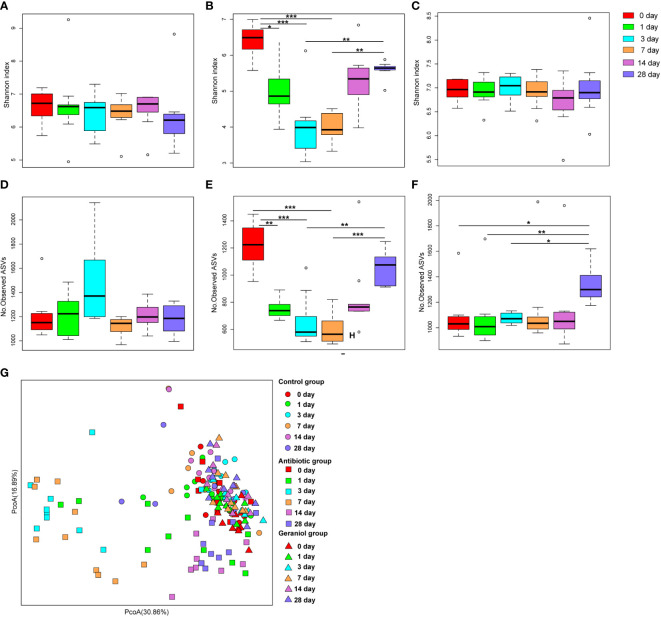
Dynamic changes of alpha and beta diversity of gut microbiotas in mice fed with saline, antibiotics and geraniol. Shannon index of gut microbiotas in mice fed with saline **(A)**, antibiotics **(B)** and geraniol **(C)**; Number of observed ASVs of gut microbiotas in mice fed with saline **(D)**, antibiotics **(E)** and geraniol **(F)**; **(G)** Principal coordinate analysis (PCoA) based on Weighted Unifrac distance of gut microbiotas in mice after being fed with saline, antibiotics and geraniol, separately. 0th means before the mice were fed with the drug; 1th day means 1 day after oral administration; 3th day means 3 days after continuous oral administration, and stopping oral administration; 7th, 14th and 28th day mean 7, 14, 28 days after oral administration, separately. (p values calculated by the One-way ANOVA test followed by *post-hoc* Dunn’s multiple comparisons test: ^∗^< 0.05, ^∗∗^< 0.01 and ^∗∗∗^< 0.001).

The Shannon index (**Figure 3B**) and the number of observed ASVs (**Figure 3E**), significantly (p < 0.05) decreased after the mice were fed with cefotaxime. The gut microbial diversity of the mice continued to decrease from the 1^st^ to the 7^th^ day after providing cefotaxime by oral gavage. The diversity was recovered on the 14^th^ day after the experiment (the 10^th^ day after stopping cefotaxime orally); however, it did not return to the normal level until the 28^th^ day. In contrast, the Shannon index (**Figure 3C**) and the number of observed ASVs (**Figure 3F**) of gut microbiota in mice did not decrease after being fed with geraniol. The alpha diversity indices were similar to that in the control mice (**Figures 3A, D**). The number of observed ASVs in the mice fed with geraniol on the 28^th^ day after the experiment was significantly higher than before (0 days; **Figure 3F**). PCoA indicated that the gut microbiota composition in the geraniol group and the control group did not change significantly during the whole experiment (0^th^ to 28^th^; p > 0.05; **Figure 3G**). However, the PCoA of the gut microbiota was significantly destroyed after being fed with cefotaxime, and it did not return to the state before administration by the 28^th^ day (24^th^ day after stopping treatment; p < 0.001; **Figure 3G**).

### Therapeutic effect of geraniol against mastitis in dairy cows

The dairy cows withmastitis showed a significant increase in SCC in the milk (**Figure S4A**). After 5 days of treatment with geraniol and antibiotics (cefotaxime and kanamycin), the SCC in the dairy cows’ milk with mastitis significantly reduced (less than 2× 10^6^ cells/mL) (**Figures S4B, C**), and the clinical symptoms subsided (Table S7). During the treatment by breast infusionof geraniol, 10 of the 13 sick cows recovered with anaverage cure time of 7 days, while 10 of the 14 sick cows recovered with antibiotics wiyh anaverage cure time of 6 days (Table S7). These results indicated that the average cure rate of geraniol was similar to that of antibiotics.

### Geraniol treatment reduces inflammation in dairy cows

**Figure 4:**
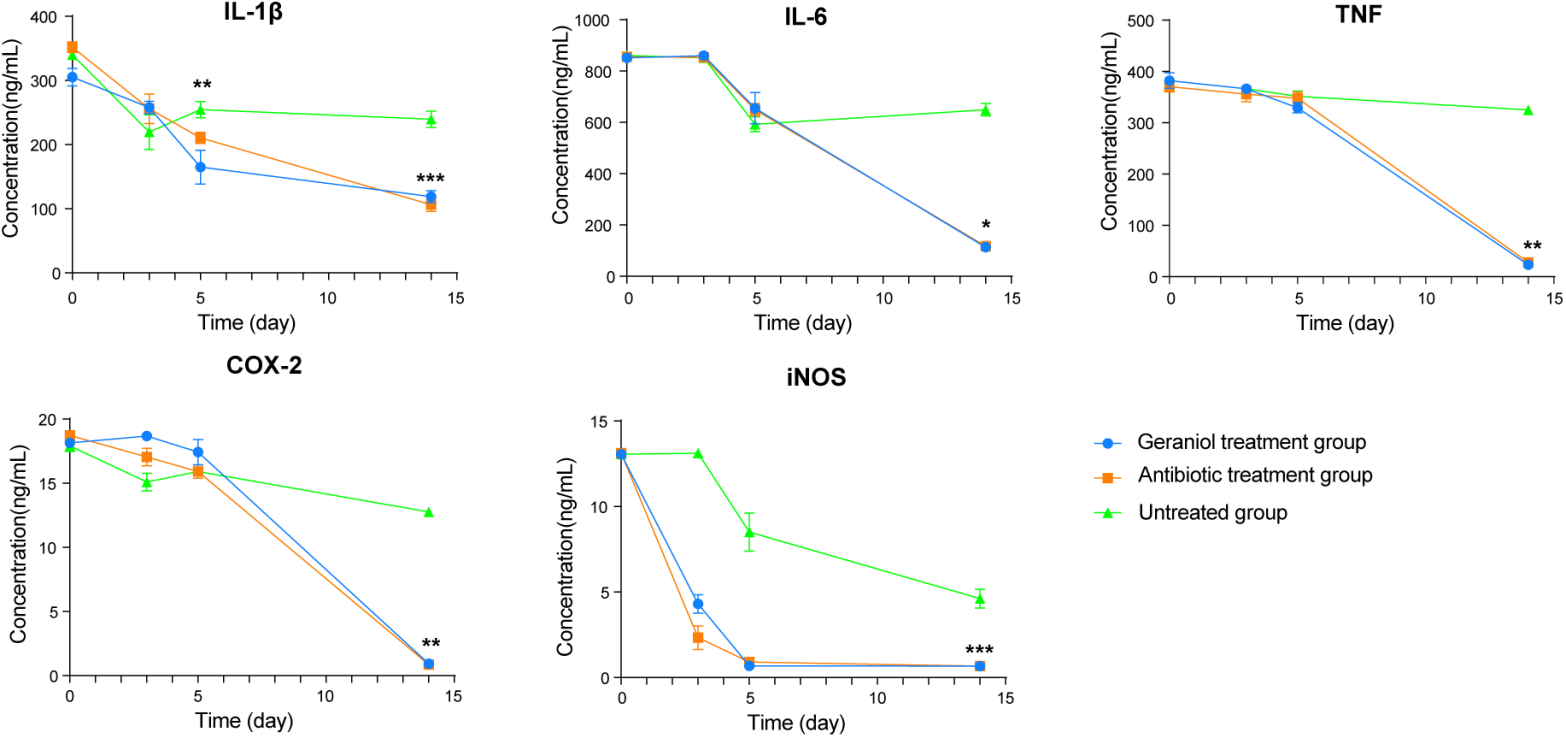
Dynamic changes of serum inflammatory factors in dairy cows with mastitis treated with antibiotics and geraniol. **(A)** Nterleukin-6; **(B)** Interleukin-1β; **(C)** Tumor necrosis factor-α; **(D)** Cyclooxygenase-2 and **(E)** Inducible nitric oxide synthase. (p values (compare with day 0) calculated by Mann–Whitney U: ^∗^< 0.05, ^∗∗^< 0.01, and ^∗∗∗^< 0.001).

During inflammation, the pro-inflammatory mediators, including IL-1β, IL-6, TNF-α, COX-2, and iNOS, are produced at the inflammation site. The levels of IL-1β, IL-6, TNF-α, COX-2, and iNOS in the serum of cows with clinical mastitis were significantly higher than those in the healthy cows (p < 0.001; **Figure S5**). The levels of these inflammatory factors (L-1β, IL -6, TNF-α, COX-2, and iNOS) in blood decreased after injecting the antibiotics and geraniol into the breast of the dairy cows with clinical mastitis (**Figure 4**). However, the levels of these inflammatory factors returned to their normal levels by the 14^th^ day after geraniol or antibiotics administration (**Figure 4**).

### Mastitis alters the milk composition and fecal bacteria in dairy cows

**Figure 5:**
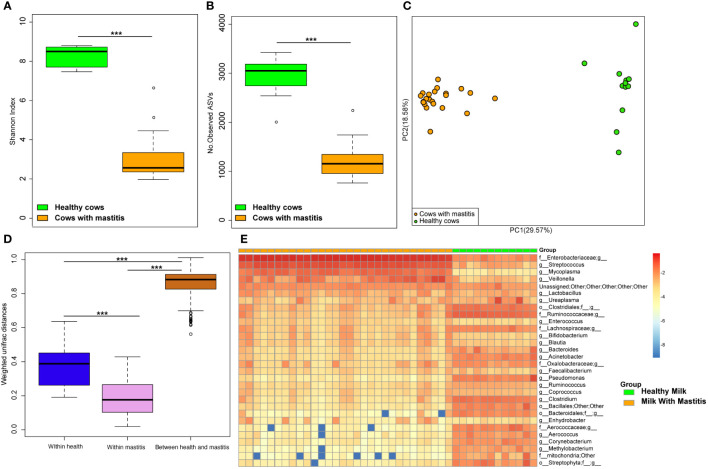
Comparison of the composition of milk bacteria between dairy cows with mastitis and healthy cows. **(A)** Shannon index and **(B)** Number of observed ASVs of milk microbiotas in dairy cows with mastitis and healthy cows; **(C)** Principal coordinate analysis (PCoA) based on Weighted Unifrac distance of milk microbiotas in dairy cows with mastitis and healthy cows; **(D)** Interindividual variations were determined by average weighted UniFrac distances between individuals in milk microbiotas of dairy cows with mastitis and healthy cows, respectively, while between-group variations were determined by distances between cows with mastitis and healthy cows. **(E)** Heatmap of top 30 most abundance genera in milk of cows with mastitis and healthy cows. (^∗∗∗^means p < 0.001; p values calculated by Mann–Whitney U in **(A, B)** p values calculated by the One-way ANOVA test followed by *post-hoc* Dunn’s multiple comparisons test in **(D)**.

**Figure 6:**
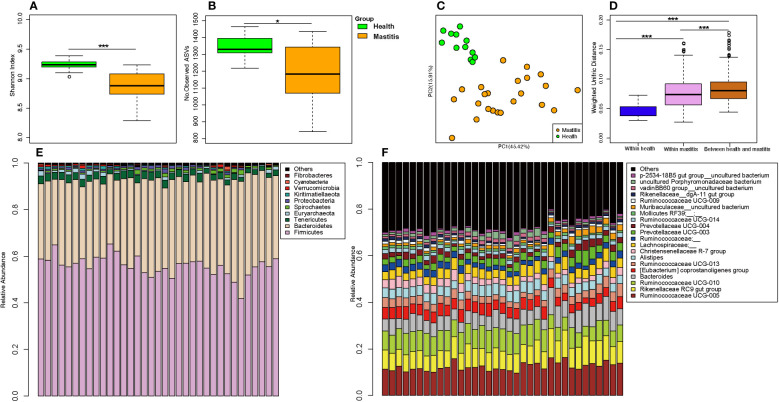
Comparison of the composition of gut microbiome between dairy cows with mastitis and healthy cows. **(A)** Shannon index and **(B)** Number of observed ASVs of gut microbiotas in dairy cows with mastitis and healthy cows; **(C)** Principal coordinate analysis (PCoA) based on Weighted Unifrac distance of gut microbiotas in dairy cows with mastitis and healthy cows; **(D)** Interindividual variations were determined by average weighted UniFrac distances between individuals in gut microbiotas of dairy cows with mastitis and healthy cows, respectively, while between-group variations were determined by distances between cows with mastitis and healthy cows. Stacked bar plots illustrate the mean relative abundance of ASVs at the phylum **(E**, top 10) and genus **(F**, top 20) level in the fecal of dairy cows with mastitis and healthy cows. (^∗<^0.05, ^∗∗∗^< 0.001; p values calculated by Mann–Whitney U in **A, B**; p values calculated by the One-way ANOVA test followed by *post-hoc* Dunn’s multiple comparisons test in **D**).

The alpha diversities of the bacteria in the milk (**Figures 5A, B**) and gut (**Figures 6A, B**) of the dairy cows with mastitis were significantly lower (p < 0.05) than those in the healthy dairy cows. PCoA indicated that the milk (**Figure 5C**) and gut (**Figure 6C**) microbiota structure of cows with mastitis was significantly different from the healthy dairy cows. Mastitis resulted in a significant increase in the intra-individual variations in milk (**Figure 5D**) and gut (**Figure 6D**) microbiota, and the phylogenetic distance between the healthy group and mastitis group was significantly larger than that in the healthy or mastitis group. The dairy cows with mastitis showed a significant imbalance of gut microbiota in milk. Mastitis significantly increased the relative abundances of *Enterobacteriaceae* (cows with mastitis, 0.546 ± 0.075; healthy cows, 0.027 ± 0.009), *Streptococcus* (cows with mastitis, 0.184 ± 0.041; healthy cows, 0.005 ± 0.004), and *Mycoplasma* (cows with mastitis, 0.072 ± 0.079; healthy cows, 0.005 ± 0.037), which was significantly lower than that of the normal bacteria in milk (**Figures 5E**), such as *Ruminococcaceae* (cows with mastitis, 0.006 ± 0.002; healthy cows, 0.085 ± 0.043), *Clostridiales* (cows with mastitis, 0.007 ± 0.004; healthy cows, 0.048 ± 0.014), *Bacillales* (cows with mastitis, 0.001 ± 0.000; healthy cows, 0.031 ± 0.022), and *Clostridium* (cows with mastitis,0.002 ± 0.001; healthy cows, 0.045 ± 0.012). However, no significant change in the relative abundance of predominant phylum (top 10) and genus (top 20) was observed between the gut microbiota of cows with mastitis and healthy cows (**Figures 6E, F**).

### Geraniol decreases the relative abundance of pathogenic bacteria and increases the relative abundance of probiotics in the milk of dairy cows with mastitis

**Figure 7:**
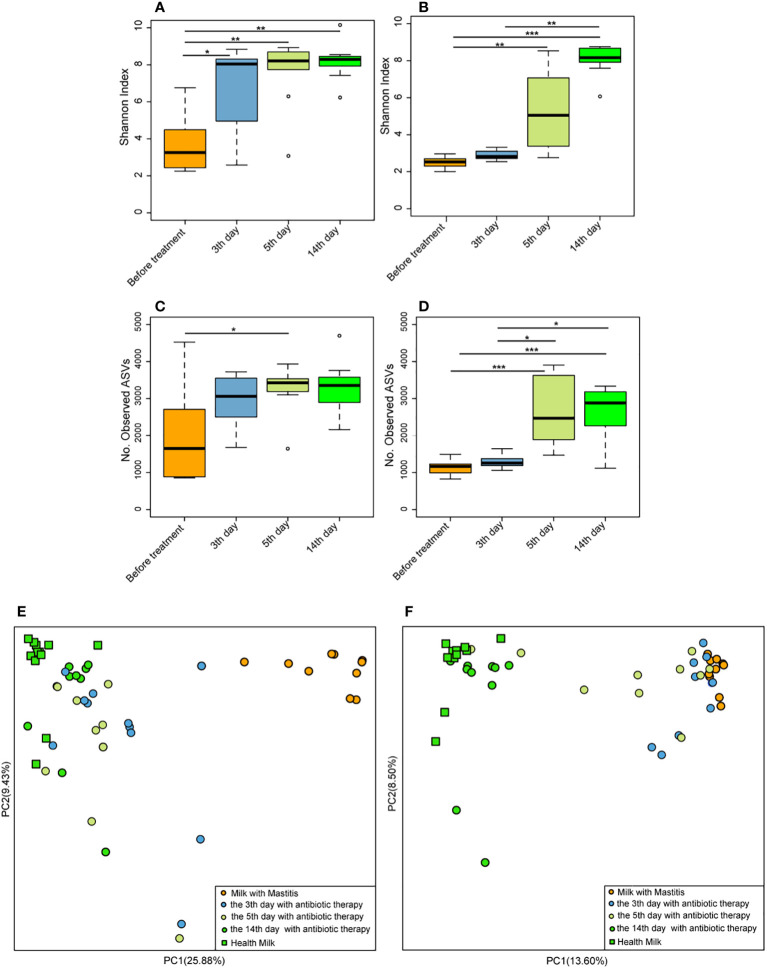
Effect of antibiotic and geraniol on milk microbiotas of dairy cows in the treatment of mastitis. Dynamic changes of Shannon index of milk microbiotas in dairy cows treated with antibiotics **(A)** andgeraniol **(B)**; Dynamic changes of Number of observed ASVs of milk microbiotas in dairy cows treated with antibiotics **(C)** andgeraniol **(D)**; Dynamic cluster of bacteria in milk of dairy cows with mastitis supported by Principal component analysis based on Weighted Unifrac distance before and after using antibiotic **(E)** and geraniol **(F)** (p values calculated by the One-way ANOVA test followed by *post-hoc* Dunn’s multiple comparisons test: ^∗^< 0.05, ^∗∗^< 0.01 and ^∗∗∗^< 0.001).

On the 3^rd^, 5^th^, and 14^th^ days after antibiotic treatment, the Shannon index of milk microbiota in the cows with mastitis was significantly higher (p < 0.05) than that of before treatment (**Figure 7A**), and the number of observed ASVs was significantly higher (p < 0.05) than that of before treatment on the 5^th^ day after antibiotic treatment (**Figure 7C**). Meanwhile, the Shannon index of milk microbiota in the cows with mastitis significantly increased (p < 0.05) on the 5^th^ and 14^th^ days after geraniol treatment (**Figure 7B**). Additionally, the number of observed ASVs was significantly higher (p < 0.05) on the 5^th^ and 14^th^ days after geraniol treatment than that of before treatment (**Figure 7D**). The antibiotic treatment group and geraniol treatment group showed significant recovery in the community structure, which was consistent with the normal milk microbiota (**Figures 7E, F**). Notably, antibiotics showed a faster recovery of milk microbiota community structure in cows with mastitis than geraniol. After three days of antibiotic treatment, the microbial community in the milk of dairy cows with mastitis was closer to the healthy dairy cows, while geraniol treatment took 14 days to restore the milk microbiota(**Figures 7E, F**).

**Figure 8:**
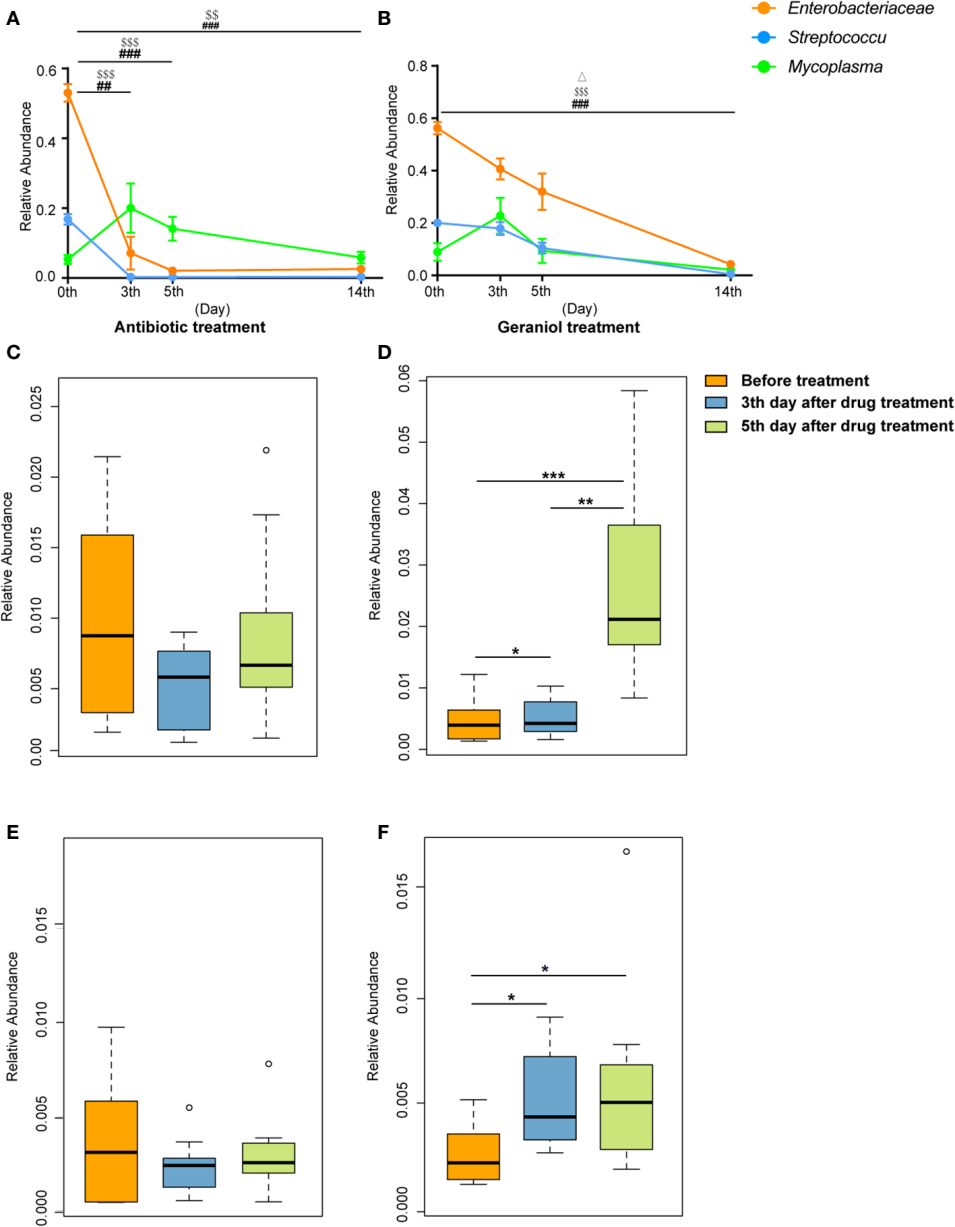
Dynamic changes of relative abundance of pathogens and probiotics in milk during the treatment of cow mastitis with antibiotics and geraniol. Dynamic changes of relative abundance of *Enterobacteriaceae*, *Streptococcus* and *Mycoplasma* in the milk of dairy cows with mastitis treated with antibiotic **(A)** and geraniol **(B)**; Dynamic changes of relative abundance of *Lactobacillus* in the milk of dairy cows with mastitis treated with antibiotic **(C)** andgeraniol **(D)** Dynamic changes of relative abundance of *Bifidobacterium* in the milk of dairy cows with mastitis treated with antibiotic **(E)** and geraniol **(F)**. 3d and 5d means after continuous administration for three and five days, separately; 14d means the 14th days after receiving drug treatment (It also means the 9th day after stopping drug treatment). [p values calculated by the One-way ANOVA test followed by *post-hoc* Dunn’s multiple comparisons test: ^∗^< 0.05, ^∗∗^< 0.01 and ^∗∗∗^< 0.001); ^##^< 0.01 and ^###^< 0.001 for *Enterobacteriaceae* in **(A)** and **(B)**; ^$$^<0.01and ^$$$^< 0.001 for *Streptococcus* in **(A, B)**, Δ< 0.05 for *Mycoplasma* in **(B)**].

The relative abundance of the pathogenic bacteria (*Enterobacteriaceae*, *Streptococcus*, and *Mycoplasma*) in milk significantly decreased following the treatment with antibiotics and geraniol (p < 0.05) (**Figures 8A, B**). In the antibiotic treatment group, the relative abundance of *Enterobacteriaceae*, *Streptococcus*, and *Mycoplasma* decreased to 0.026 ± 0.006, 0.003 ± 0.001, and 0.06 ± 0.005, respectively, on the 14^th^ day after treatment (**Figure 8A**), while it decreased to 0.04 ± 0.01, 0.004 ± 0.002, and 0.02 ± 0.01, respectively, in the geraniol treatment group (**Figure 8B**). The relative abundance of probiotics, including *Lactobacillus* and *Bifidobacterium*, in the milk of cows with mastitis significantly increased (p < 0.05) with geraniol treatment (3^rd^ and 5^th^ days; **Figures 8D, F**); however, the relative abundance of these probiotics did not increase significantly in the antibiotic treatment group (**Figures 8C, E**).

Similarly, the quantitative PCR analysis results were consistent with the 16S rRNA sequencing results showing similar changing patterns for the number of pathogens (**Figure S6**) and probiotics (**Figure S7**) in the cows’ milk in the geraniol treatment group and antibiotic treatment group.

### Gut microbiota in cows remain safe during mastitis treatment with geraniol

**Figure 9:**
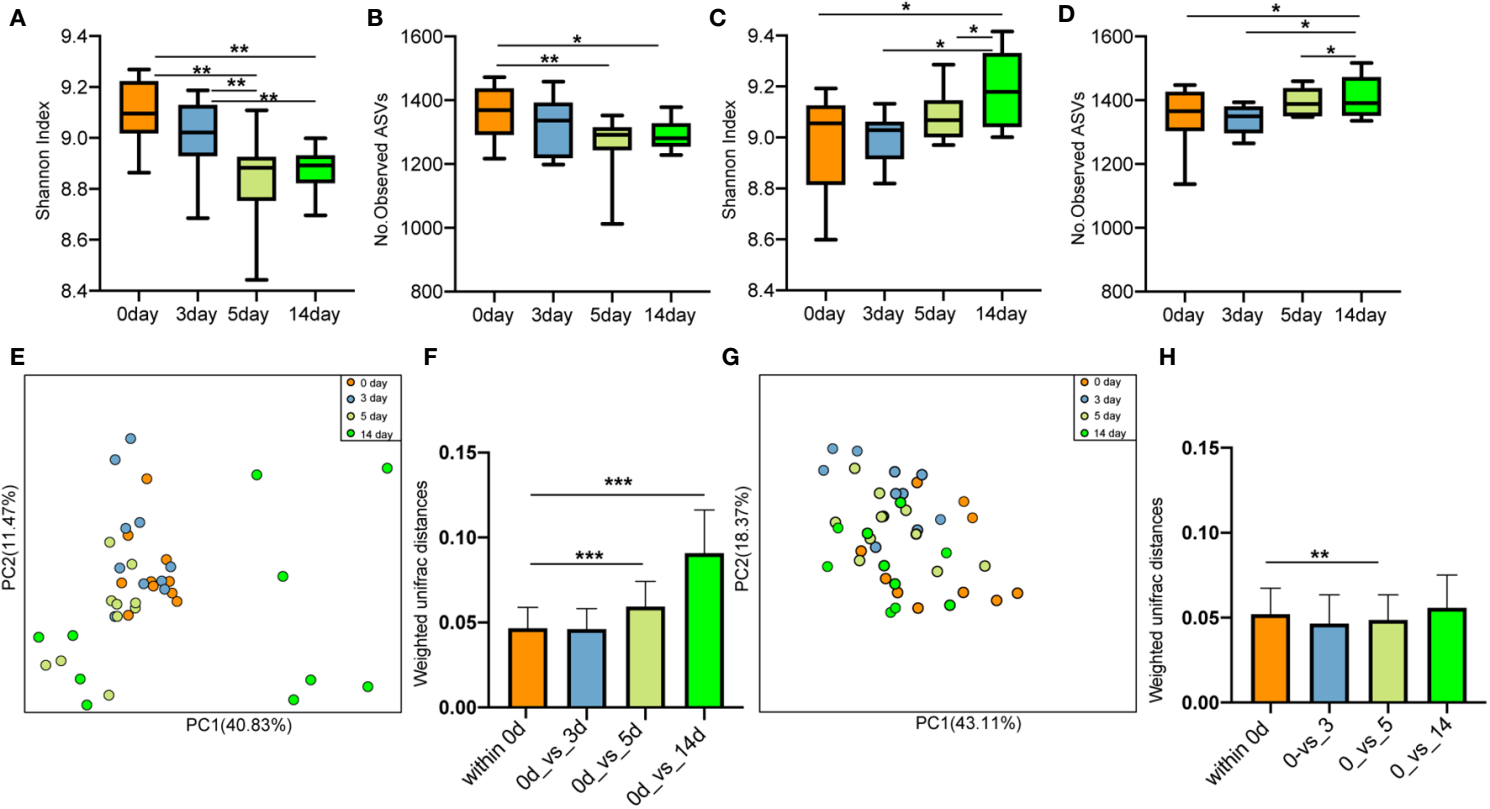
Effect of antibiotic and geraniol on gut microbiotas of dairy cows in the treatment of mastitis. Dynamic changes of **(A)** Shannon index and **(B)** Number of observed ASVs of gut microbiotas in dairy cows treated with antibiotics; Dynamic changes of **(C)** Shannon index and **(D)** Number of observed ASVs of gut microbiotas in dairy cows treated with geraniol; Dynamic cluster of bacteria in milk of dairy cows with mastitis supported by Principal component analysis based on Weighted Unifrac distance before and after using antibiotic **(E)** and geraniol **(G)**; Phylogenetic distance of gut microbiotas in cows after administration of antibiotics **(F)** geraniol **(H)** and before administration. utot-hoc Dunn’s multiple comparisons test: ^∗^< 0.05, ^∗∗^< 0.01 and ^∗∗∗^< 0.001).

After antibiotic injection, the Shannon index and the number of observed ASVs of gut microbiota in the cows with mastitis descreated, showing no recovery until the 14^th^ day (10 days after discontinuing antibiotics) (**Figures 9A, B**). Conversely, the Shannon index (**Figure 9C**) and the number of observed ASVs (**Figure 9D**) increased steadily after treatment with geraniol. On the 14^th^ day (10^th^ day after stopping geraniol) after geraniol treatment, the Shannon index and the number of observed ASVs of gut microbiota in the cows with clinical mastitis were significantly higher (p <0.05) than before treatment.

Furthermore, the PCoA based on weighted Unifrac distance showed that antibiotic treatment significantly destroyed the gut microbiota structure of cows with mastitis (**Figure 9E**). On the 10^th^ day after discontinuing antibiotic treatment (14^th^ day), the gut microbiota structure showed the largest variation than that of before treatment. The phylogenetic distance between the gut microbiota after (5^th^/14^th^ day) and before treatment was significantly larger (p < 0.05) than the samples before treatment (**Figures 9F**). However, geraniol did not destroy the gut microbiota community structure in the cows with mastitis during treatment (**Figure 9G**). Compared with the samples before treatment (the 0^th^ day), the phylogenetic distance in the gut microbiota of cows with mastitis show no significant increase between the 3^th^/5^th^/14^th^ days after receiving geraniol treatment and before treatment (p > 0.05) (**Figure 9H**)

### Geraniol has a short residual time in cow’s milk

A total of 140 milk samples were collected from 20 cured cows (each cow was sampled once a day for seven consecutive days) of the geraniol treatment group (n = 10) and antibiotic treatment group (n = 10) to detect the drug residues in milk. Cephalexin and kanamycin remained in the milk of cured cows on the 7^th^ day after cephalexin and kanamycin injection (**Table 1**). Meanwhile, the quantitative analysis of the antibiotic residues indicated that metabolism could only reduce the residue dose of antibiotics by 0.1 to 0.4 times daily, without any reduction in cephalexin and kanamycin residues in the milk of five cows within two or three days. No geraniol residue was detected in the cows’ milk on the 4^th^ day after stopping geraniol treatment (**Table 2**). Furthermore, the quantitative analysis of the drug residues showed that the geraniol residues decreased 6 times day by day after stopping geraniol.

**Table 1:** Residue of cephalexin and kanamycin in milk of dairy cows after stopping to antibiotic.

Cow ID	Days after stopping the drug						
	Day 1	Day 2	Day 3	Day 4	Day 5	Day 6	Day 7
CA_1	+/*	+/*	+/*	+/*	+/*	+/*	+/*
CA_2	+/*	+/*	+/*	+/*	+/*	+/*	+/*
CA_3	+/*	+/*	+/*	+/*	+/*	+/*	+/*
CA_4	+/*	+/*	+/*	+/*	+/*	+/*	+/*
CA_5	+/*	+/*	+/*	+/*	+/*	+/*	+/*
CA_6	+/*	+/*	+/*	+/*	+/*	+/*	+/*
CA_7	+/*	+/*	+/*	+/*	+/*	+/*	+/*
CA_8	+/*	+/*	+/*	+/*	+/*	+/*	+/*
CA_9	+/*	+/*	+/*	+/*	+/*	+/*	+/*
CA_10	+/*	+/*	+/*	+/*	+/*	+/*	+/*

“+” means that the residue of cephalexin can be detected in milk; “*” means that the residue of kanamycin be detected in milk.

**Table 2:** Residue of geraniol in milk of dairy cows after stopping to inject geraniol.

Cow ID	Days after stopping the drug						
	Day 1	Day 2	Day 3	Day 4	Day 5	Day 6	Day 7
CG_1	+	+	+	–	–	–	–
CG_2	+	+	+	+	–	–	–
CG_3	+	+	+	–	–	–	–
CG_4	+	+	+	–	–	–	–
CG_5	–	–	–	–	–	–	–
CG_6	+	+	+	–	–	–	–
CG_7	+	+	+	–	–	–	–
CG_8	+	–	–	–	–	–	–
CG_9	+	–	–	–	–	–	–
CG_10	+	–	–	–	–	–	–

“+” means that the residue of geraniol can be detected in milk; “-” means that the residue of geraniol cannot be detected in milk.

The reduction in cefalexin and kanamycin injection dose did not change its residual time in the dairy cows. These two antibiotics were detected in the milk of cured cows on the 10th day after injecting a low dose (2 g) of cephalexin and kanamycin into the cow’s breast (**Table S8**).

### Geraniol retains antibacterial activity and results in less pathogen resistance

**Figure 10:**
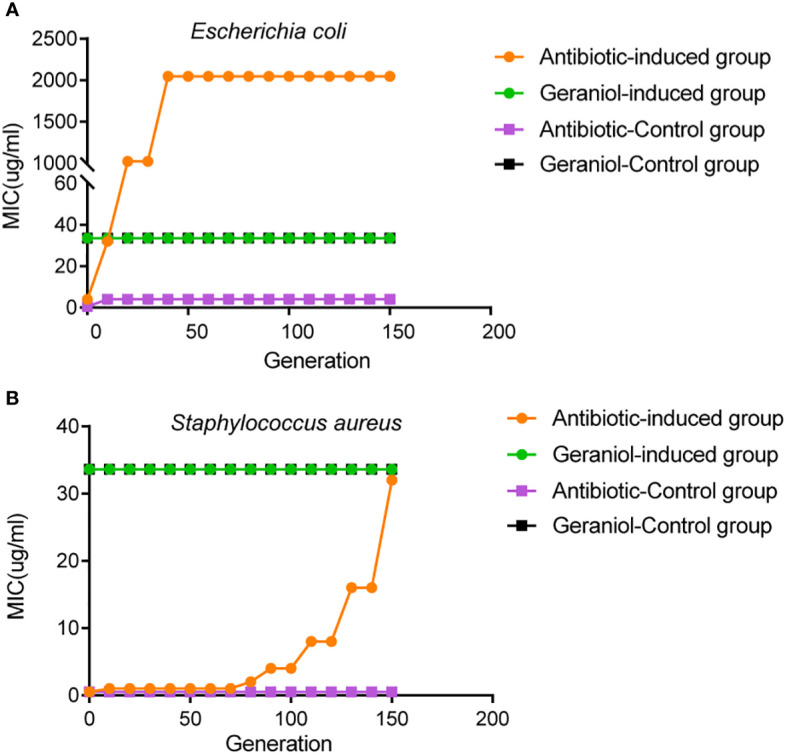
Effects of drug resistance of bacteria to geraniol and amoxicillin *via* passage cultivation with sub-inhibitory concentrations of drug. **(A)** Dynamic changes of MIC value of geraniol to the *Escherichia coli* strain ATCC25922 from 1-150 generations of subculture at 1/2MIC. **(B)** Dynamic changes of MIC value of amoxicillin to the *Staphylococcus aureus* strain ATCC25923 from 1-150 generations of subculture 1/2MIC.

The initial MIC values of geraniol and amoxicillin for the *Escherichia coli* strain ATCC25922 were 33.6 µg/mL and 4 µg/mL, respectively. The initial MIC values of geraniol and ampicillin for the *Staphylococcus aureus* strain ATCC25923 were 33.6 µg/mL and 0.5 µg/mL, respectively. The MIC value of geraniol for *Escherichia coli* and *Staphylococcus aureus* did not change after being induced by sub-inhibitory concentration for 150 generations (**Figures 10A, B**). The MIC value of amoxicillin for *Escherichia coli* ATCC25922 increased eight times (32 µg/mL) by the 10^th^ generation, 256 times (1024 µg/mL) by the 20^th^ generation, and 512 times (2048 µg/mL) by the 40^th^ to 150th generations (**Figure 10A**), and the MIC value of ampicillin for *Staphylococcus aureus* increased two times (1.0 µg/mL) by the 10th generation, 4 times (2.0 µg/mL) by the 80th generations, 8 times (4.0 µg/mL) by the 90th generations, 16 times (8.0 µg/mL) by the 110th generations, 32 times (16.0 µg/mL) by the 130th generations, and 62 times (32.0 µg/mL) by the 150th generation (**Figure 10B**). This result suggested that prolonged use of geraniol might not induce bacterial resistance.

## Discussion

In the present study, geraniol reduced the inflammation of the mammary glands in cows with clinical mastitis, which was consistent with the previous reports on anti-inflammatory and antioxidant effects of geraniol on the Metabolic Syndrome (MetS) rat model (Ibrahim et al., 2015). So far, the studies on geraniol anti-inflammatory properties have mainly focused on mouse model (Bachiega and Sforcin, 2011; Ibrahim et al., 2015; Ye et al., 2019). To the best of our knowledge, this is the first study to report the effect of geraniol in reducing inflammatory factors in dairy cows with a naturally occurring disease. Although the disease cure rate with geraniol was similar to antibiotics, geraniol took longer to cure dairy cows with clinical mastitis, indicating the differences between the mechanism of action in traditional Chinese medicine and antibiotics. Generally, antibiotics act against infectious diseases by directly killing the pathogenic bacteria, whereas traditional Chinese medicines exert anti-infectious properties through comprehensive actions (Tan and Vanitha, 2004), such as inhibiting or killing the pathogenic bacteria (Asif, 2012; Meng et al., 2017) (usually not as good as antibiotics), immune regulation (Ma et al., 2013; Shi et al., 2020), and anti-inflammatory activity (Pan et al., 2011). As such, geraniol showed a prolonged course of treatment.

Furthermore, both geraniol and antibiotics restored the milk microbiota in cows with mastitis. The diversity of milk microbiota in cows with mastitis significantly improved after breast perfusion treatment with antibiotics or geraniol. Meanwhile, the proportion of pathogenic bacteria (*Enterobacteriaceae*, *Streptococcus*, and *Mycoplasma*) in the milk of cows with mastitis was as high as 80%, resulting in a low diversity. Treatment with antibiotics or geraniol effectively inhibited these pathogenic bacteria, quickly restoring the milk microbiota diversity. Studies using a third-generation antibiotic, cephalosporin, to treat cow mastitis have confirmed that antibiotics can significantly improve microbial diversity (Ganda et al., 2016), which is consistent with the present study results. Compared with antibiotics, geraniol reduced the relative abundance of pathogenic bacteria and restored the mastitis-induced structural damaged of milk microbiota upon a prolonged treatment. This slow action might be attributed to the TCM characteristics. These results suggest that geraniol could be used to treat cow mastitis more mildly. In a previous study, geraniol significantly improved the relative abundance of *Lactobacillus* and *Bifidobacterium*, the common probiotics in farm animals known for enhancing the immunity of dairy cows, increasing milk yield, and reducing pathogenic bacteria during livestock breeding (Paliy et al., 2020; Markowiak and Śliżewska, 2018). Nagahata et al. reported that the intramammary infusion of *Bifidobacterium breve* eliminated the pathogens and decreased the SCC in dairy cows with chronic subclinical mastitis (Nagahata et al., 2020); it also enhanced the innate immune response of mammary glands in the lactating dairy cows (Nagahata et al., 2020). In a previous study, feeding with *Lactobacillus casei* and *Lactobacillus plantarum* improved the quality and quantity of cow milk production (Xu et al., 2017). Therefore, it can be inferred that geraniol inhibits pathogenic bacteria and increases the abundance of probiotics, showing its potential to maintain long-term health and milk yield in dairy cows with mastitis. So far, several herbs (*Black Cumin*, *Curcuma zeodharia*, *Curcuma mangga*, and *Curcuma aeruginosa*) have been reported to significantly increase milk yield, milk protein content, and milk lactose content in cows with subclinical mastitis and significantly decrease the occurrence of mastitis (Nurdin et al., 2011). However, no antibiotic has been reported to demonstrate such a characteristic to date. Therefore, further studies are highly necessitated to verify the contribution of an increased abundance of probiotics in milk toward anti-infection and maintainingmilk yield in dairy cows.

In the dairy industry, the treatment of bovine clinical mastitis is mainly based on the extensive use of antibiotics, causing the microbiota in the rumen and rectum of cows to change significantly (Pol and Ruegg, 2007). Notably, the microbial community structure could not be restored for a long time (> 18 days) after withdrawing antibiotics (Ji et al., 2018). In the present study, the community structure of the intestinal microflora in dairy cows and mice was severely damaged after treatment with antibiotics, which could not return to the normal level within the analyzed time frame. The gut microbiome is necessary and highly beneficial for the host, as they can help in development (Schwarzer et al., 2018), harvesting energy (Savage, 1977), defending pathogens (Kamada et al., 2013), and regulating immunity (Round and Mazmanian, 2009). Therefore, treating dairy cows with antibiotics might lead to the deterioration of their health status.An experimental Chinese medicinal formula alleviated ulcerative colitis (UC) symptoms in the model mice by restoring the function and diversity of gut microbiota (Pan et al., 2020). Similarly, geraniol did not destroy the gut microbial community structure and significantly increased the gut microbiome diversity in dairy cows unlick antibiotics.

Furthermore, antibiotic residues were detected in the milk of dairy cows for a prolonged period (> 7 days) after withdrawing the antibiotics, exerting a significant impact on the health of dairy cows and environmental safety. Several studies have identified and reported antibiotic residues in milk after18 days of treatment (Jones, 1999). Antibiotic residues have been reported to destroy the gut microbiome and affect the health of calves (Pereira et al., 2018). However, discarding the milk with antibiotic residues leads to huge economic losses for the dairy industry, causing antibiotic pollution to the environment (Jayalakshmi et al., 2017). Meanwhile, geraniol residues in milk were detected for a short time. Geraniol can be metabolized in animals (Chadha and Madyastha, 1984), with a half-life of about 12.5 ± 1.5 min (Pavan et al., 2018). The half-lives of cephalexin and kanamycin ranged from 0.6 to 1.2 h and 0.575 to 1.527 h, respectively (Henley et al., 1996; Thrupp, 2003). This might explain the shorter residual time of geraniol in cows’ milk than that of cephalexin and kanamycin. Moreover, the compound does not pose a threat to the host (Pavan et al., 2018). Besides, the *in vitro* antimicrobial experiments showed that antibiotics induced bacterial resistance, while geraniol hardly caused resistance. This characteristic is more common in traditional Chinese medicines, probably due to the mechanisms responsible for antibacterial properties (Rodriguez-Garcia et al., 2017). Therefore, studying the specific mechanisms underlying the anti-infective property of geraniol is of great significance. Overall, the above results confirm that geraniol, a single enolate compound obtained from Chinese herbal medicine, such as *Fructus Tsaoko* or easily available through chemical synthesis (Takabe et al., 1977), could be used as a potential alternative to antibiotics and a cost-effective treatment option for mastitis.

## Data availability statement

The datasets presented in this study can be found in online repositories. The names of the repository/repositories and accession number(s) can be found below:PRJNA747729 (SRA).

## Ethics statement

The animal study was reviewed and approved by Experimental Animal Welfare Ethics Committee of Chengdu Medical College. Written informed consent was obtained from the owners for the participation of their animals in this study.

## Author contributions

MD and WG designed this study. WG, MQ, and MD wrote this manuscript. WG, MD, FS, and MQ analyzed the data. ZP, MQ, WG, and NL performed the laboratory experiments. KR, RN, SZ, LL, LG and YZ collected the samples. MY provided suggestions for manuscript writing. All authors contributed to the article and approved the submitted version.

## Glossary

**Table:**

FEMA	American Spice and Extract Manufacturers Association
FDA	the U.S. Food and Drug Administration
SPF	pecific pathogen-free
KM	Kunming
LB	Luria-Bertani
MLD	minimum lethal dose
LD50	half lethal dose
E. coli	Escherichia coli
ED50	50% effective dose
WBC	white blood cell count
LYM	absolute lymphocyte value
MID	absolute intermediate cell
GRA	absolute granulocyte value
RBC	red blood cell count
HGB	hemoglobin
HCT	hematocrit
MCV	mean red blood cell volume
MCH	mean hemoglobin content
MHCH	mean hemoglobin concentration
PLT	platelet count
PCT	platelet packed volume
MPV	average platelet volume
SCC	Somatic cell counts
IL-6	interleukin-6
IL-1&beta;	interleukin-1&beta;
TNF-&alpha;	tumor necrosis factor-&alpha;
COX-2	cyclooxygenase-2
iNOS	inducible nitric oxide synthase
ELISA	enzyme linked immunosorbent assay
PCR	polymerase chain reaction
GC-MS	gas chromatography-mass spectrometer system
MHB	Mueller-Hinton Broth
MIC	minimum inhibitory concentration
PCoA	principal coordinate analysis
MetS	Metabolic Syndrome
UC	alleviating Ulcerative colitis
