# Supplementary material for: Geraniol-a potential alternative to antibiotics for bovine mastitis treatment without disturbing the host microbial community or causing drug residues and resistance
Source: Front Cell Infect Microbiol. 2023 Feb 16;13:1126409. doi: 10.3389/fcimb.2023.1126409 (PMC9978373; doi:10.3389/fcimb.2023.1126409)
Supplement: Supplementary file 1 [file DataSheet_1.pdf]

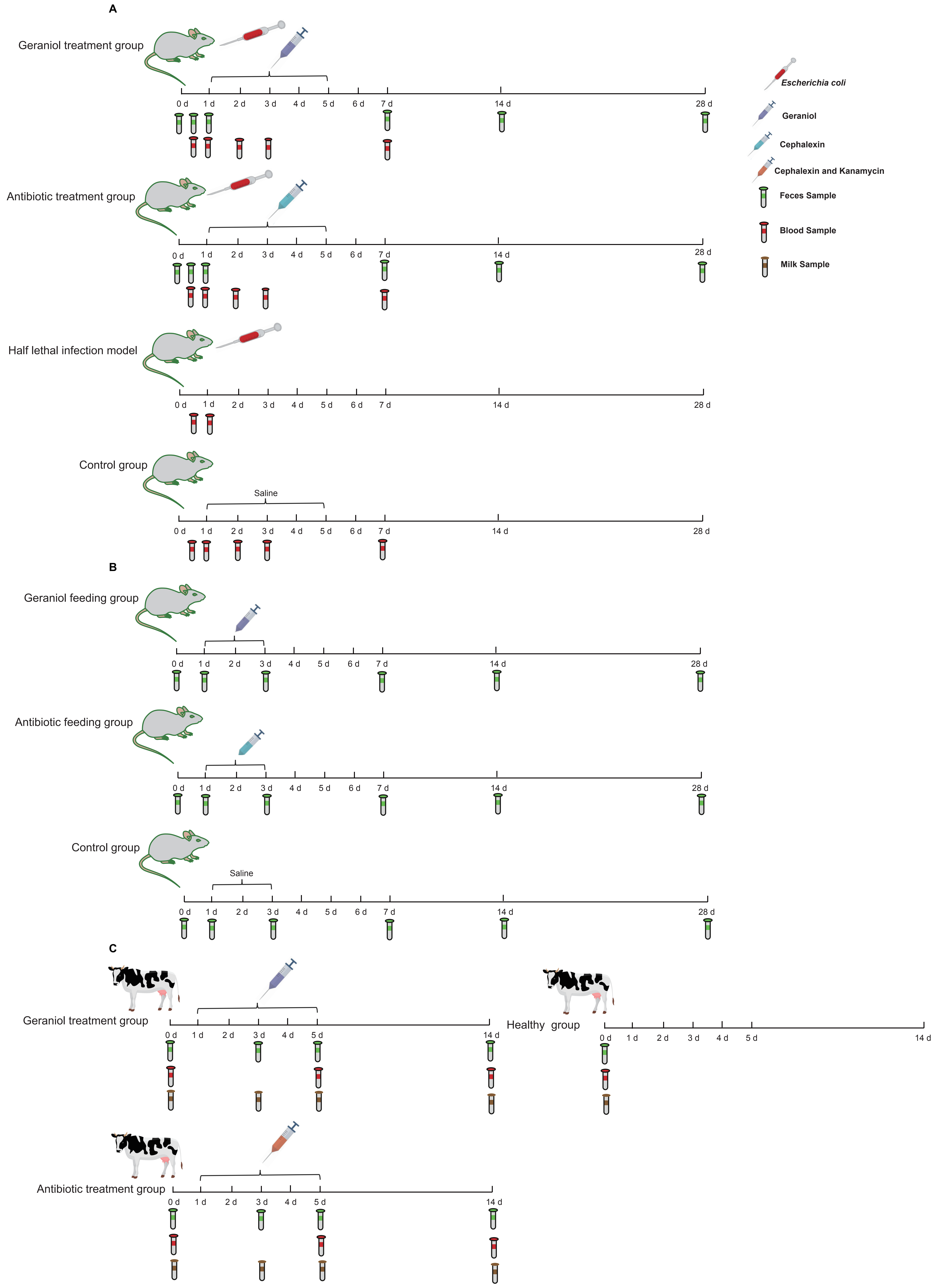

Fig. S1. Schematic diagram of the animal experiment. (A) Cefotaxime and geraniol anti-bacterial infection experiment in model mice; (B) Experiment of oral administration of cefotaxime and geraniol in mice; (C) Experiment of antibiotic and geraniol treatment of cow mastitis.

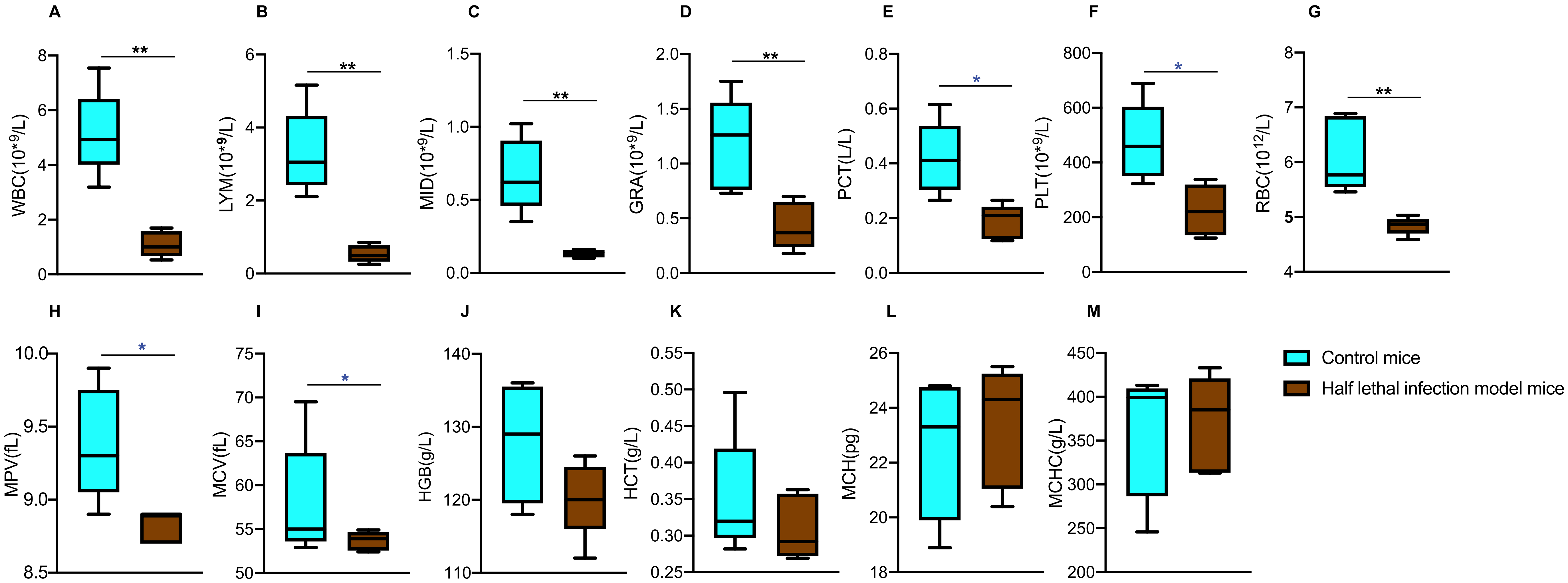

Fig. S2. Effect of pathogenic *Escherichia coli* infection on Blood Routine Indexes in mice. (A) white blood cell count (WBC); (B) absolute lymphocyte value (LYM); (C) absolute intermediate cell (MID); (D) absolute granulocyte value (GRA); (E) platelet packed volume (PCT); (F) platelet count (PLT); (G) red blood cell count (RBC); (H) average platelet volume (MPV); (I) mean red blood cell volume (MCV); (J) hemoglobin (HGB); (K) hematocrit (HCT); (L) mean hemoglobin content (MCH); (M) mean hemoglobin concentration(MHCH). (p values calculated by Mann–Whitney U: * < 0.05 and ** < 0.01)

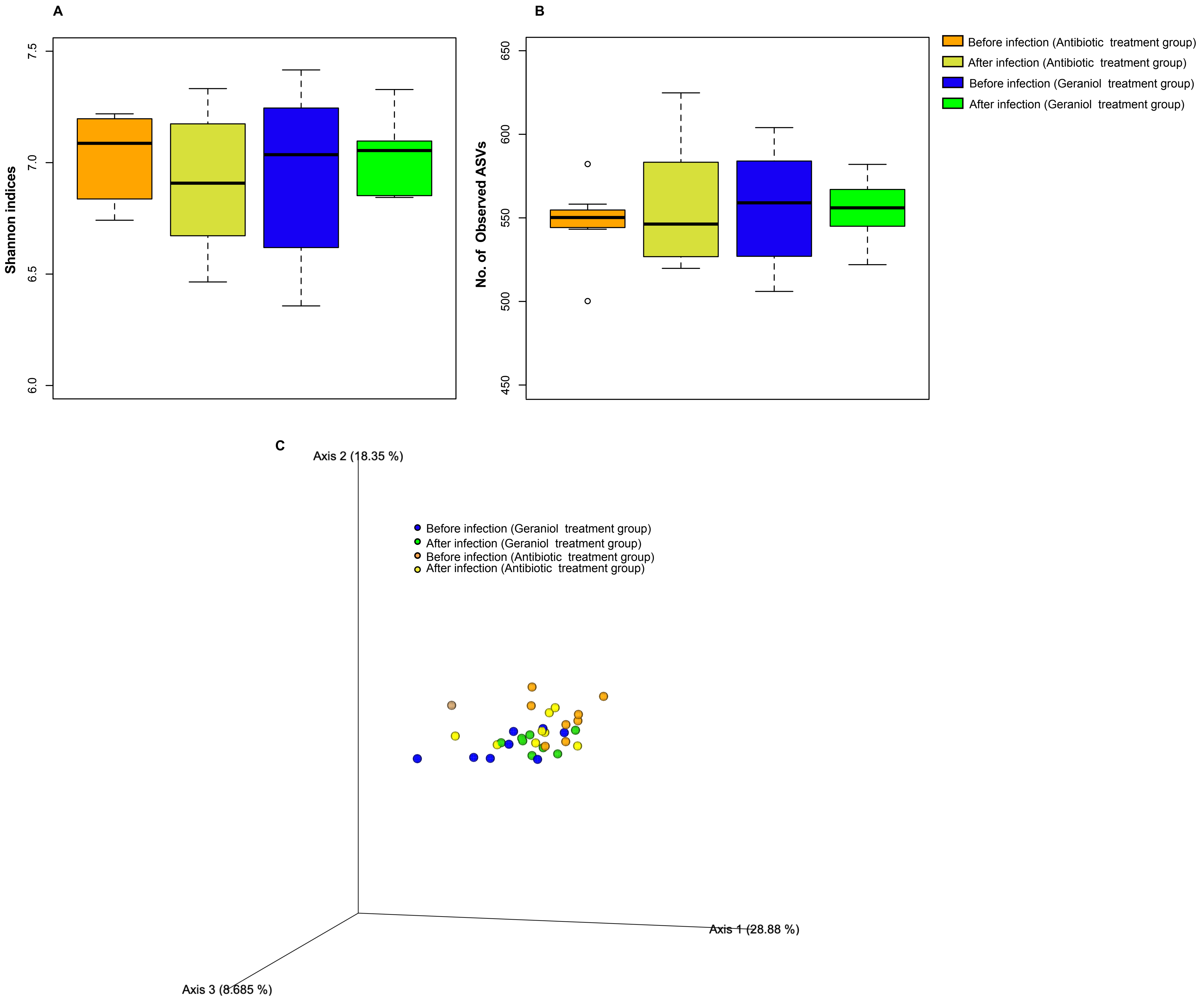

Fig. S3. Comparative analysis of gut microbiotas diversity in mice infected with half lethal dose (LD50) of *Escherichia coli* before and after infection. The alpha diversity measured by Shannon index (A) and number of observe OTUs (B); Principal coordinate analysis (PCoA) based on Weighted Unifrac distance (C).

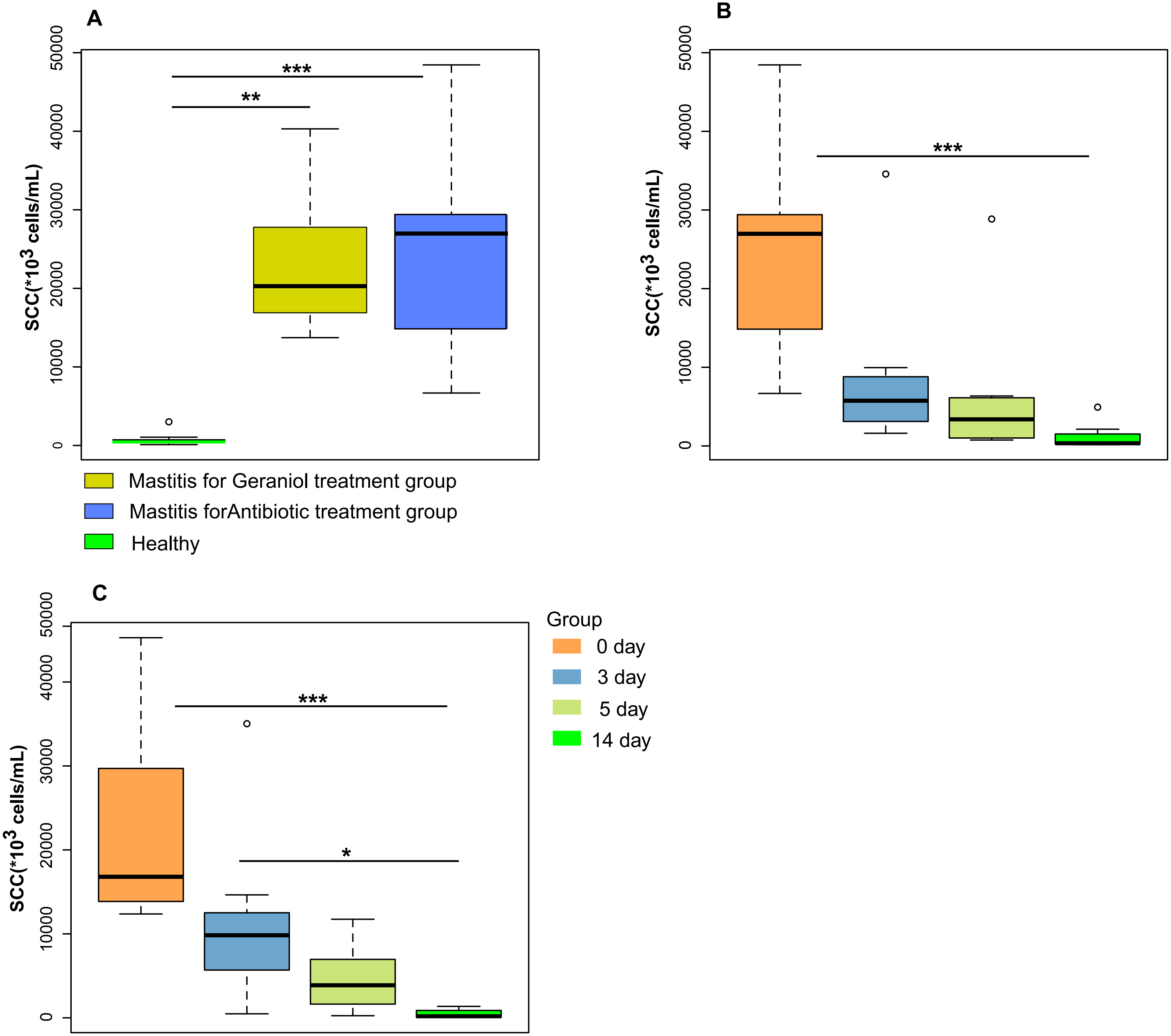

Fig. S4. The number of somatic cells in the milk of cows. (A) Comparison of the number of somatic cells in milk between healthy and cows with mastitis; Dynamic changes of somatic cells in dairy cows with mastitis treated with antibiotics (B) and geraniol (C). (p values calculated by the One-way ANOVA test followed by post-hoc Dunn's multiple comparisons test: * < 0.05, ** < 0.01 and *** < 0.001)

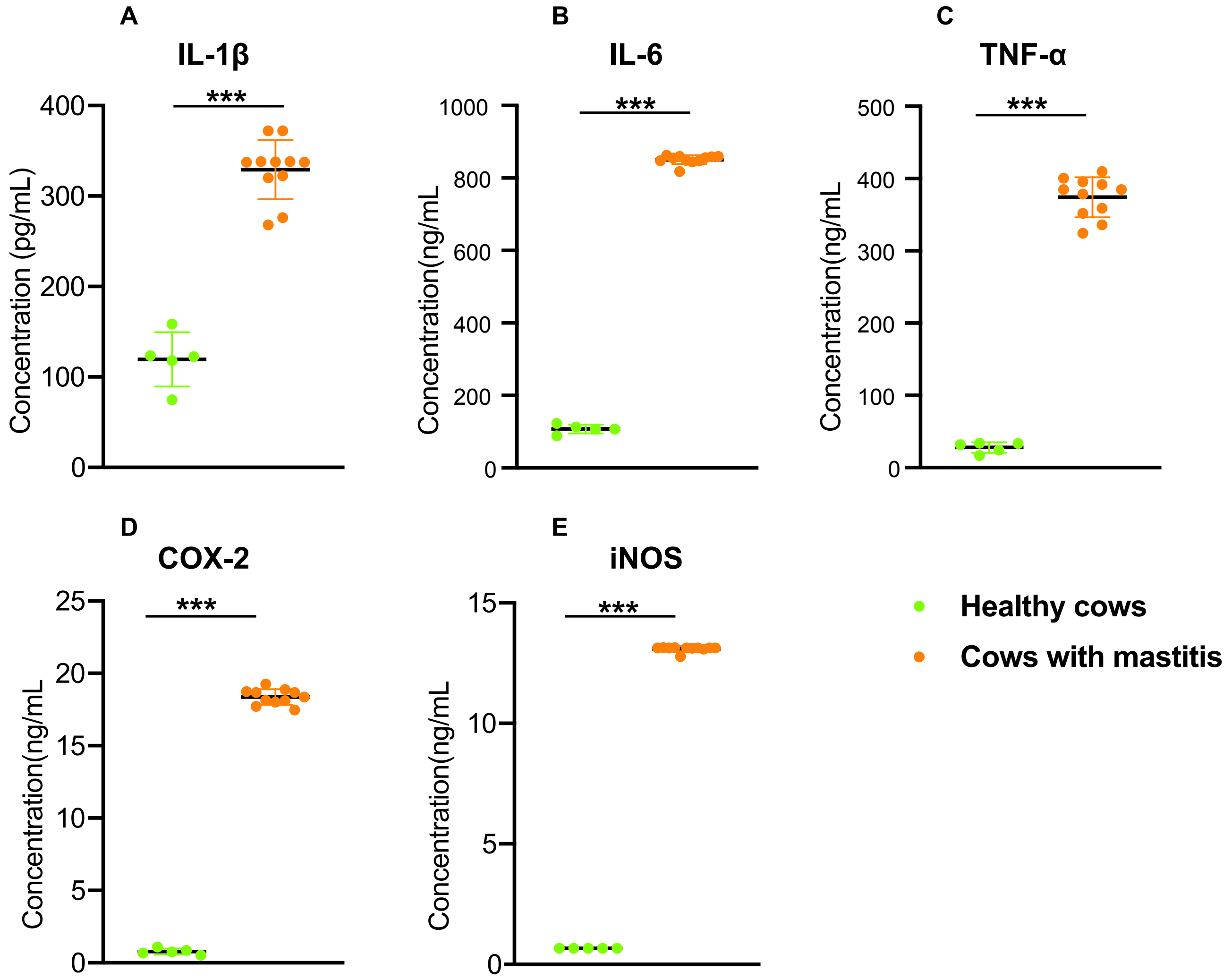

Fig. S5. Comparison of serum inflammatory factors between cows with clinical mastitis and healthy cows. (A) Nterleukin-6; (B) Interleukin-1β; (C)Tumor necrosis factor-α; (D) Cyclooxygenase-2 and (E) Inducible nitric oxide synthase. *** means p <0.001 by Mann-Whitney U test.

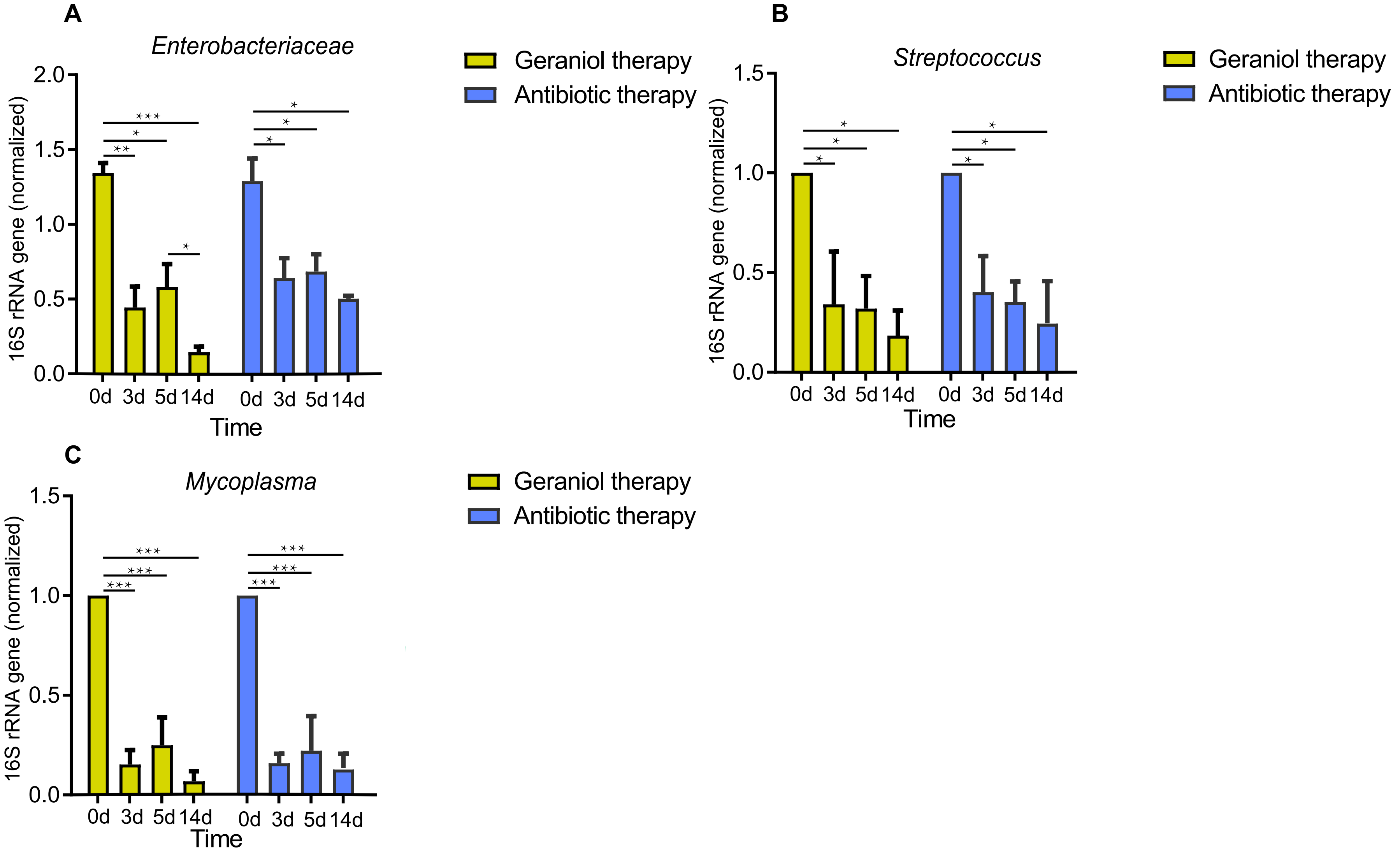

Fig. S6. Quantitative polymerase chain reaction (PCR) estimates the abundance of pathogens in the milk of dairy cows. (A) *Enterobacteriaceae*; (B) *Streptococcus*; (C) *Mycoplasma*. (p values calculated by the Oneway-ANOVA test followed by post-hoc Dunn's multiple comparisons test: * $p < 0.05$, ** $p < 0.01$, *** $p < 0.001$)

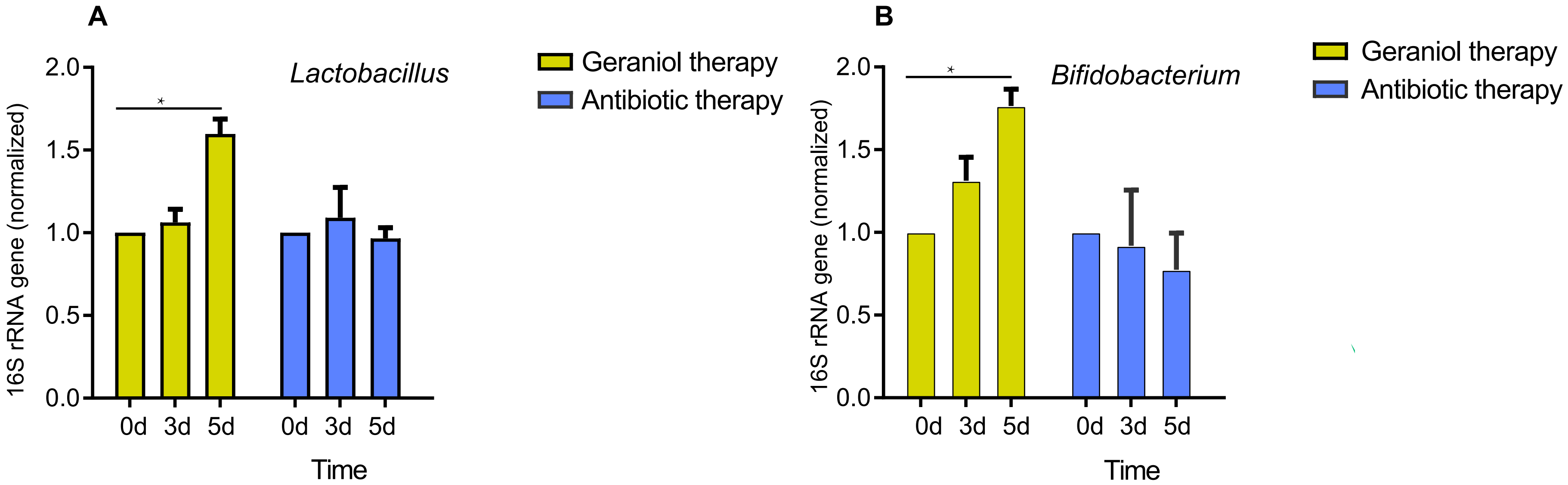

Fig. S7. Quantitative polymerase chain reaction (PCR) estimates the abundance of probiotics in the milk of dairy cows. (A) *Lactobacillus*; (B) *Bifidobacterium*. (p values calculated by the One-way ANOVA test followed by post-hoc Dunn's multiple comparisons test: * $p < 0.05$)
