# Supplementary material for: Geraniol-a potential alternative to antibiotics for bovine mastitis treatment without disturbing the host microbial community or causing drug residues and resistance
Source: Front Cell Infect Microbiol. 2023 Feb 16;13:1126409. doi: 10.3389/fcimb.2023.1126409 (PMC9978373; doi:10.3389/fcimb.2023.1126409)
Supplement: Supplementary file 2 [file DataSheet_2.pdf]

Table S1. Detailed information of cows in this study.

SampleID	Age(month)	Variety	Health condition	Location	Treatment group
H_1	66	Holstein-Friesian	Healthy	Qingbaijiang District, Chengdu	Ccontrol
H_2	75	Holstein-Friesian	Healthy	Qingbaijiang District, Chengdu	Ccontrol
H_3	65	Holstein-Friesian	Healthy	Qingbaijiang District, Chengdu	Ccontrol
H_4	53	Holstein-Friesian	Healthy	Qingbaijiang District, Chengdu	Ccontrol
H_5	47	Holstein-Friesian	Healthy	Qingbaijiang District, Chengdu	Ccontrol
H_6	60	Holstein-Friesian	Healthy	Qingbaijiang District, Chengdu	Ccontrol
H_7	64	Holstein-Friesian	Healthy	Qingbaijiang District, Chengdu	Ccontrol
H_8	57	Holstein-Friesian	Healthy	Qingbaijiang District, Chengdu	Ccontrol
H_9	87	Holstein-Friesian	Healthy	Qingbaijiang District, Chengdu	Ccontrol
H_10	64	Holstein-Friesian	Healthy	Qingbaijiang District, Chengdu	Ccontrol
H_11	59	Holstein-Friesian	Healthy	Qingbaijiang District, Chengdu	Ccontrol
H_12	72	Holstein-Friesian	Healthy	Qingbaijiang District, Chengdu	Ccontrol
CA_1	62	Holstein-Friesian	Mastitis	Qingbaijiang District, Chengdu	Antibiotic
CA_2	60	Holstein-Friesian	Mastitis	Qingbaijiang District, Chengdu	Antibiotic
CA_3	85	Holstein-Friesian	Mastitis	Qingbaijiang District, Chengdu	Antibiotic
CA_4	50	Holstein-Friesian	Mastitis	Qingbaijiang District, Chengdu	Antibiotic
CA_5	66	Holstein-Friesian	Mastitis	Qingbaijiang District, Chengdu	Antibiotic
CA_6	62	Holstein-Friesian	Mastitis	Qingbaijiang District, Chengdu	Antibiotic
CA_7	48	Holstein-Friesian	Mastitis	Qingbaijiang District, Chengdu	Antibiotic
CA_8	58	Holstein-Friesian	Mastitis	Qingbaijiang District, Chengdu	Antibiotic
CA_9	98	Holstein-Friesian	Mastitis	Qingbaijiang District, Chengdu	Antibiotic
CA_10	54	Holstein-Friesian	Mastitis	Qingbaijiang District, Chengdu	Antibiotic
CA_11	55	Holstein-Friesian	Mastitis	Qingbaijiang District, Chengdu	Antibiotic
CA_12	88	Holstein-Friesian	Mastitis	Qingbaijiang District, Chengdu	Antibiotic
CA_13	95	Holstein-Friesian	Mastitis	Qingbaijiang District, Chengdu	Antibiotic
CA_14	65	Holstein-Friesian	Mastitis	Qingbaijiang District, Chengdu	Antibiotic
CG_1	84	Holstein-Friesian	Mastitis	Qingbaijiang District, Chengdu	Geraniol
CG_2	86	Holstein-Friesian	Mastitis	Qingbaijiang District, Chengdu	Geraniol
CG_3	62	Holstein-Friesian	Mastitis	Qingbaijiang District, Chengdu	Geraniol
CG_4	74	Holstein-Friesian	Mastitis	Qingbaijiang District, Chengdu	Geraniol
CG_5	86	Holstein-Friesian	Mastitis	Qingbaijiang District, Chengdu	Geraniol
CG_6	61	Holstein-Friesian	Mastitis	Qingbaijiang District, Chengdu	Geraniol
CG_7	47	Holstein-Friesian	Mastitis	Qingbaijiang District, Chengdu	Geraniol
CG_8	60	Holstein-Friesian	Mastitis	Qingbaijiang District, Chengdu	Geraniol
CG_9	71	Holstein-Friesian	Mastitis	Qingbaijiang District, Chengdu	Geraniol
CG_10	62	Holstein-Friesian	Mastitis	Qingbaijiang District, Chengdu	Geraniol
CG_11	85	Holstein-Friesian	Mastitis	Qingbaijiang District, Chengdu	Geraniol
CG_12	63	Holstein-Friesian	Mastitis	Qingbaijiang District, Chengdu	Geraniol
CG_13	74	Holstein-Friesian	Mastitis	Qingbaijiang District, Chengdu	Geraniol

Table S2. Quantitative PCR primers for *Enterobacteriaceae*, *Streptococcus*, *Mycoplasma*, *Lactobacillus* and *Bifidobacterium*.

**Bacteria**	**Forward primer**	**Reverse primer**
Enterobacteriaceae	CTGGCAAGCTTGAGTCTCGT	CTGAGCGTCAGTCTTCGTCC
Streptococcus	TGAGTGCAGAAGGGGAGAGT	GAGCCTCAGCGTCAGTTACA
Mycoplasma	GGCGTAAAGCGTCTGTAGGT	CGCATTTCACCGCTTCACAA
Bifidobacterium	CGTGTAGTCCTGGTAGCGTG	CGATGGACTTTCACACCGGA
Lactobacillus	ATGTGAAAGCCTTCGGCTCA	TCTACGCATTCCACCGCTAC

Table S3. The thermocycling parameters of Quantitative PCR primers for *Enterobacteriaceae*, *Streptococcus*, *Mycoplasma*, *Lactobacillus* and *Bifidobacterium*.

Number of cycles	Enterobacteriaceae and Streptococcus		*Mycoplasma and Lactobacillus*		*Bifidobacterium*	
	Temperature	Time	Temperature	Time	Temperature	Time
1	95℃	15min	95℃	15min	95℃	15min
40	95℃	10s	95℃	10s	95℃	10s
	57℃	20s	55℃	20s	57.5℃	20s
	72℃	30s	72℃	30s	72℃	30s
1	65℃	5s	65℃	5s	65℃	5s

Table S4. Minimum lethal dose (MLD) and median lethal dose (LD50) of *Escherichia coli* in mice.

Group	Number of mice	Bacteria solution dosage （CFU/kg）	Number of deaths	Mortality (%)
Control	10	-	0	0
1	10	5.85×10^{7}	10	100
2	10	2.925×10^{7}	10	100
3	10	3.663×106	9	90
4	10	2.75×10^{6}	8	80
5	10	1.831×10^{6}	6	60
6	10	1.371×10^{6}	5	50
7	10	0.911×10^{6}	5	50

Table S5. Comparison of different doses of geraniol in prevention of *Escherichia coli* infection with minimum lethal dose in mice.

Group	Medicine	Dose （g/kg）	Number of mice	Survival	Survival rate (%)
Control	Normal saline	/	10	10	100
Model	Normal saline	/	10	0	0
Solvent	Twain-80	/	10	0	0
Positive control	Cefotaxime	0.30	10	10	100
Experimental group 1	Geraniol	0.261	10	10	100
Experimental group 2	Geraniol	0.166	10	7	70
Experimental group 3	Geraniol	0.0944	10	5	50
Experimental group 4	Geraniol	0.07	10	2	20
Experimental group 5	Geraniol	0.045	10	3	30

Table S6. Comparison of curative effects of geraniol (ED50) and antibiotics on mice infected with half lethal dose (LD50) of *Escherichia coli*.

Group	Medicine	Bacteria solution dosage（CFU/kg）	Dose（g/kg）	Number of mice	Survival	Survival rate (%)
Control	Saline	0.911×10^6 (Inactivated)	/	10	10	100
Half lethal model	Saline	0.911×10^6	/	10	5	50
Antibiotic treatment	Cefotaxime	0.911×10^6	0.30	10	10	100
Geraniol treatment	Geraniol	0.911×10^6	0.261	10	10	100

Table S7. Therapeutic effect of antibiotics and geraniol on mastitis in dairy cows.

Sample ID	Age (month)	Clinical symptoms	Treatment drug	Administration method	Results of t reatment	Healing time(day)	Improvement of symptoms
CA_1	62	Breast is mildly swollen, milk is grayish-white and flocculent	Cefotaxime and Kanamycin	Breast perfusion	Cured	3	Swelling subsided, the milk returned to milky-white
CA_2	60	The whole breast area is moderate swollen, milk is grayish-white and forms clots	Cefotaxime and Kanamycin	Breast perfusion	Cured	4	Swelling subsided, the milk returned to normal
CA_3	85	Breast swelling, the milk is yellow-white and sticky	Cefotaxime and Kanamycin	Breast perfusion	Cured	3	Swelling almost subsided, the milk returned to milky-white without abnormality
CA_4	50	Breast is mildly swollen, the milk produce flocculent	Cefotaxime and Kanamycin	Breast perfusion	Cured	5	Swelling subsided completely, the milk is milky white without abnormality
CA_5	66	The whole breast area is red, swollen and hard, producing yellow sticky milk	Cefotaxime and Kanamycin	Breast perfusion	Cured	5	The breast returned to normal, the milk returned to milky-white without abnormality
CA_6	62	The milk is dark yellow, sticky and purulent	Cefotaxime and Kanamycin	Breast perfusion	Cured	4	The milk returned to milky-white without abnormality
CA_7	48	Part areas of the breast swelling, roduce dark yellow and purulent milk	Cefotaxime and Kanamycin	Breast perfusion	Cured	5	Swelling subsided, the milk returned to normal
CA_8	58	Produce flocculent milk	Cefotaxime and Kanamycin	Breast perfusion	Cured	4	Flocculent disappeared and milk retured to milky-white
CA_9	98	Produce flocculent milk	Cefotaxime and Kanamycin	Breast perfusion	Cured	7	Flocculent disappeared and milk retured to milky-white
CA_10	54	Produce dark yellow and purulent milk	Cefotaxime and Kanamycin	Breast perfusion	Cured	5	Flocculent disappeared and milk retured to milky-white
CA_11	55	The whole breast area is red, swollen and hard, producing yellow sticky milk	Cefotaxime and Kanamycin	Breast perfusion	Not cured	NA	It has been producing milky flocculent milk
CA_12	88	Breast swelling, the milk is yellow-white and sticky	Cefotaxime and Kanamycin	Breast perfusion	Not cured	NA	Breast firmness, roduce flocculent milk (Cull)
CA_13	95	The whole breast area is moderate swollen, milk is grayish-white and forms clots	Cefotaxime and Kanamycin	Breast perfusion	Not cured	NA	Breast firmness, roduce flocculent milk (Cull)
CA_14	65	The whole breast area is red, swollen and hard, producing yellow sticky milk	Cefotaxime and Kanamycin	Breast perfusion	Not cured	NA	The symptoms did not improve (Cull)
CG_1	84	Breast is mildly swollen, the milk produce flocculent	Geraniol	Breast perfusion	Cured	6	Swelling subsided, the milk returned to normal
CG_2	86	The whole breast area is red and swollen, producing yellow sticky milk	Geraniol	Breast perfusion	Cured	7	Swelling subsided, the milk returned to normal
CG_3	62	Breast is mildly swollen, the milk produce flocculent	Geraniol	Breast perfusion	Cured	6	Swelling subsided, the milk returned to normal
CG_4	74	The milk produce flocculent	Geraniol	Breast perfusion	Cured	5	Flocculent disappeared in milk, the milk returned to normal

CG_5	86	Produce flocculent milk	Geraniol	Breast perfusion	Cured	7	Flocculent disappeared in milk, the milk returned to normal
CG_6	61	Milk is grayish-white and flocculent	Geraniol	Breast perfusion	Cured	7	The milk returned to milky-white without abnormality
CG_7	47	The whole breast area is red, swollen and hard, producing yellow sticky milk	Geraniol	Breast perfusion	Cured	5	Flocculent disappeared in milk, the milk returned to normal
CG_8	60	Milk is grayish-white and flocculent	Geraniol	Breast perfusion	Cured	6	The milk returned to milky-white without abnormality
CG_9	71	Produce dark yellow and purulent milk	Geraniol	Breast perfusion	Cured	5	The milk returned to milky-white without abnormality
CG_10	62	Part areas of the breast swelling, roduce dark yellow and purulent milk	Geraniol	Breast perfusion	Cured	4	Swelling subsided, the milk returned to normal
CG_11	85	Breast mass, produce flocculent milk	Geraniol	Breast perfusion	Not cured	NA	The symptoms did not improve (Cull)
CG_12	63	Breast mass, produce flocculent milk	Geraniol	Breast perfusion	Not cured	NA	The symptoms did not improve (Cull)
CG_13	74	Breast is mildly swollen, the milk produce flocculent	Geraniol	Breast perfusion	Not cured	NA	The symptoms did not improve (Cull)

Table S8. Residue of cephalexin and kanamycin in milk of dairy cows after stopping therapy with a low dose.

Cow ID / Days after infection	CA_15	CA_16	CA_17	CA_18	CA_19
Day 1	+/*	+/*	+/*	+/*	+/*
Day 2	+/*	+/*	+/*	+/*	+/*
Day 3	+/*	+/*	+/*	+/*	+/*
Day 4	+/*	+/*	+/*	+/*	+/*
Day 5	+/*	+/*	+/*	+/*	+/*
Day 6	+/*	+/*	+/*	+/*	+/*
Day 7	+/*	+/*	+/*	+/*	+/*
Day 8	+/*	+/*	+/*	+/*	+/*
Day 9	+/*	+/*	+/*	+/*	+/*
Day 10	+/*	+/*	+/*	+/*	+/*

"+" means that the residue of cephalexin can be detected in milk; "*" means that the residue of kanamycin be detected in milk.
